# Characterization of Extremely Drug-Resistant and Hypervirulent *Acinetobacter baumannii* AB030

**DOI:** 10.3390/antibiotics9060328

**Published:** 2020-06-17

**Authors:** Manu Singh, P. Malaka De Silva, Yasser Al-Saadi, Jacek Switala, Peter C. Loewen, Georg Hausner, Wangxue Chen, Ismael Hernandez, Santiago Castillo-Ramirez, Ayush Kumar

**Affiliations:** 1 Department of Microbiology, University of Manitoba, Winnipeg, MB R3T 2N2, Canada; singhm12@myumanitoba.ca (M.S.); desilpmp@myumanitoba.ca (P.M.D.S.); alsaadiy@myumanitoba.ca (Y.A.-S.); Jacek.Switala@umr.umanitoba.ca (J.S.); Peter.Loewen@umanitoba.ca (P.C.L.); Georg.Hausner@umanitoba.ca (G.H.); 2 Human Health Therapeutics, National Research Council Canada, 100 Sussex Drive, Ottawa, ON K1A 0R6, Canada; Wangxue.Chen@nrc-cnrc.gc.ca; 3 Programa de Genómica Evolutiva, Centro de Ciencias Génomicas, Universidad Nacional Autónoma de México, Cuernavaca 62210, Mexico; ishernan@ccg.unam.mx (I.H.); iago@ccg.unam.mx (S.C.-R.)

**Keywords:** multidrug resistance, virulence, gram-negative, comparative genomics, insertion elements

## Abstract

*Acinetobacter baumannii* is an important nosocomial bacterial pathogen. Multidrug-resistant isolates of *A. baumannii* are reported worldwide. Some *A. baumannii* isolates display resistance to nearly all antibiotics, making treatment of infections very challenging. As the need for new and effective antibiotics against *A. baumannii* becomes increasingly urgent, there is a need to understand the mechanisms of antibiotic resistance and virulence in this organism. In this work, comparative genomics was used to understand the mechanisms of antibiotic resistance and virulence in AB030, an extremely drug-resistant and hypervirulent strain of *A. baumannii* that is a representative of a recently emerged lineage of *A. baumannii* International Clone V. In order to characterize AB030, we carried out a genomic and phenotypic comparison with LAC-4, a previously described hyper-resistant and hypervirulent isolate. AB030 contains a number of antibiotic resistance- and virulence-associated genes that are not present in LAC-4. A number of these genes are present on mobile elements. This work shows the importance of characterizing the members of new lineages of *A. baumannii* in order to determine the development of antibiotic resistance and virulence in this organism.

## 1. Introduction

*Acinetobacter baumannii* infections are being reported with increasing frequency worldwide [1,2]. *A. baumannii*’s success as an important hospital-acquired pathogen is attributed to its ability to acquire and accumulate genes associated with antibiotic resistance. As a result, multidrug-resistant isolates of *A. baumannii* are commonly reported from various clinical settings [3,4,5]. Recently, the WHO categorized carbapenem-resistant *A. baumannii* as the top priority pathogen against which treatment options are urgently needed [6]. Understanding the mechanisms of resistance as well as how antibiotic resistance genes spread in clinical isolates of *A. baumannii* is critical for finding new and effective treatment options for antibiotic-resistant *A. baumannii* isolates. With recent advances in next-generation sequencing, comparative genomics has emerged as a powerful tool for understanding the mechanisms of antibiotic resistance and virulence in bacterial pathogens [7,8]. 

Recently, a lineage sequence type 758 (ST758) of *A. baumannii* belonging to the International Clone V has been described [9]. Isolates belonging to IC5 lineage have been reported from clinical settings in North and Central America [9], underscoring the need to characterize the isolates belonging to this lineage. *A. baumannii* AB030, an extremely drug-resistant (XDR) isolate, was one of the first members described from this lineage [10]. Due to its XDR phenotype as well as it being a member of recently emerging lineage, it was undertaken to characterize AB030 to gain insights into its antibiotic resistance and virulence mechanisms. Using a combination of comparative genomic and phenotypic approaches, genetic factors that contribute to its antibiotic resistance and virulence of AB030 were determined. The genome sequence of AB030 was compared to that of LAC-4, a previously described multidrug-resistant and hypervirulent isolate of *A. baumannii* [11]. Following comparative genomics analysis, phenotypic assays were carried out to interpret and confirm the in silico predictions. 

## 2. Results and Discussion

A recent analysis [9] shows that AB030 is a member of a recently emerged lineage (ST758) belonging to the international clone V. AB030, isolated in Canada, shares a common ancestry with eight other isolates described from Mexico [9], indicating that ST758 is perhaps spreading in hospitals in the Americas. As such, characterization of members of ST758 is important to understand genotypic and phenotypic profiles of this lineage. We used LAC-4 for the comparison of AB030’s virulence and antibiotic resistance. LAC-4, although a member of ST10 that clusters separately from AB030, is a hyper-resistant and hypervirulent *A. baumannii* strain [11] and therefore serves as a good reference strain to determine the antibiotic resistance and virulence potential of AB030.

### 2.1. Genome Analysis

AB030’s chromosome is larger with 4,335,793 bp, compared to 3,954,354 bp in LAC-4 (Table 1). No plasmids were detected in AB030. LAC-4 has been previously shown to contain two plasmids [11]. 

**Figure 1:**
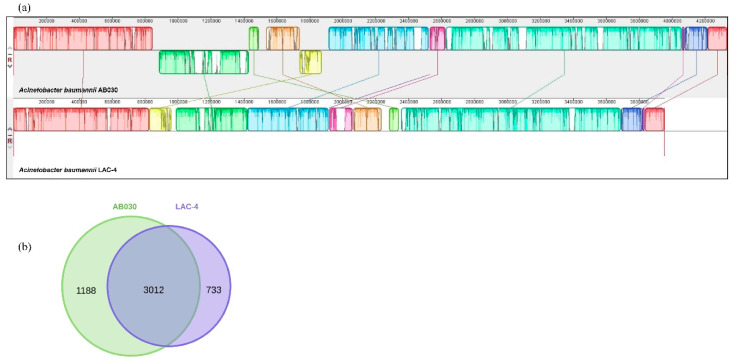
Whole genome alignment showing genomic rearrangements between the genomes of AB030 (top) and LAC-4 (bottom) (**a**); showing shared or unique CDS between AB030 and LAC-4 (**b**).

Progressive Mauve alignment on these chromosomes (Figure 1a) highlights genomic rearrangements between these two strains and highlights the regions that add to the size of the genome of AB030 in comparison to LAC-4. Some of these differences are described later in this section.

### 2.2. Common Coding Sequences in AB030 and LAC-4

**Figure 2:**
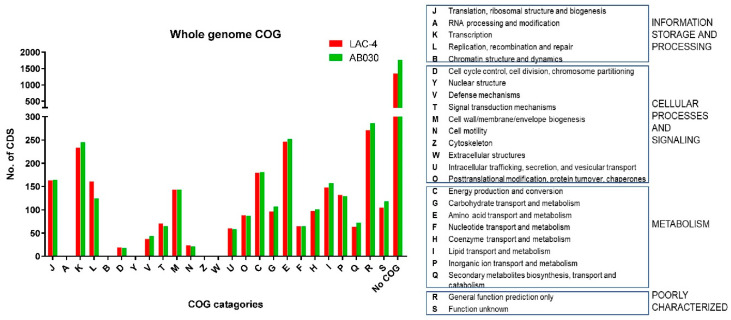
Functional categorization of the whole genomes of LAC-4 and AB030 using COG database.

The AB030 genome contains 4200 CDS whereas LAC-4 contains 3745 CDS. Of these, 3012 CDS are common in AB030 and LAC-4 at ≥95% identity, while 1188 and 733 CDS are unique for AB030 and LAC-4, respectively (Figure 1b). Comparisons of functional categories according to the COG database for all CDS in these genomes are shown in Figure 2. The majority of these unique sequences are classified as either hypothetical proteins or phage elements. Amino acid sequence searches in the NCBI database of hypothetical proteins resulted in no significant hits (E-value = 1 × 10^6^). The COG categories show that AB030 has more (1764) functionally uncharacterized CDS than LAC-4 (1346). 

### 2.3. Genomic Islands, Phage Sequences, and Insertion Sequences

Antibiotic resistance of *A. baumannii* is often attributed to its ability to readily acquire resistance genes present in mobile DNA elements [12]. We analyzed and compared the presence of genomic islands, phage elements, and insertion sequences in AB030 and LAC-4.

Genomic islands (GIs) are large (>10 kb) genetic elements that contribute to fitness and adaptation of bacteria to various environmental conditions [13]. They can also carry genes for antibiotic resistance (resistance islands) as well as virulence (pathogenic islands) and therefore contribute to the rapid evolution of bacterial pathogens such as *A. baumannii*. Twelve different GIs have been described in LAC-4 [11]. The AB030 genome was analyzed for the presence of these 12 GIs. A summary of GIs from LAC-4 and their presence in AB030 is provided in Table 2 as well as in Figure S1a–l. Of the 12 reported GIs in LAC-4, GI1 (Figure S1a) is the only one that contains antibiotic resistance genes, those for streptomycin (*strA* and *strB*) and sulphonamide (*sul*2) resistance [11]. All of the genes present in this genomic island were 100% conserved in AB030, albeit the island in AB030 also contained two additional genes, *cmlA* and *ISAba43*. The rest of the genomic islands were present only in parts with the exception of GI2, which contained a 15-gene copper resistance cluster [11] in LAC-4 (Table 2 and Figure S1b) and GI8, which was completely missing from AB030. GI3 has been shown to contain AdeIJK efflux pump in LAC-4 [11]. This pump, which is constitutively expressed in *A. baumannii*, is also present in AB030 (Figure S1c). Overall, our data show that the GIs present in LAC-4 are largely absent in AB030.

Genomic island AbaR is considered a potential contributor to the multidrug-resistant (MDR) phenotype of *A. baumannii* [14]. It was first reported as a large island (86 kb) harboring multiple antibiotic resistance genes [15] including those that confer resistance to streptomycin, aminoglycosides, tetracycline, and chloramphenicol [16]. A subsequent study showed that AbaR genomic island is common among *A. baumannii* isolates and is present in two-thirds of all *A. baumannii* genomes sequenced [17]. Many variants of AbaR-type resistance islands are known to exist with most containing three or four resistance genes with a median length of 17.63 kb [17]. AbaR-type islands have a strong preference for the *comM* gene (ATP-dependent protease) in *A. baumannii* [18,19], however, the *comM* gene in both AB030 and LAC-4 did not contain any insertions. Since, on rare occasions, AbaR insertion can occur at other sites as well [17], we analyzed both genomes for the presence of genes (*orf1*, *tniA*, *tniB*, *orf2*, *orf3*) that constitute the backbone of AbaR. None of these five genes were present in AB030 or LAC-4. Therefore, our analysis shows that AbaR is not present in either of the two strains. 

**Figure 3:**
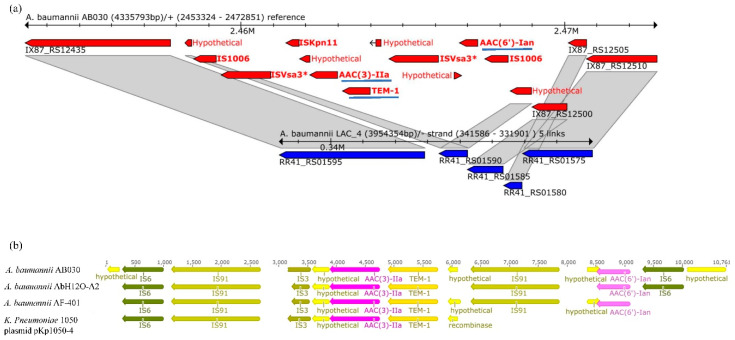
Presence of the ~9 Kb resistance island present in AB030 genome (red) (**a**). This island, missing in LAC-4 (blue), contains two aminoglycoside resistance genes and a β-lactam resistance gene (underlined). Comparison of the ~9 kb resistance island found in AB030 with those reported in A. baumannii and K. pneumoniae clinical isolates (**b**).

We also carried out an analysis to determine GIs unique to AB030 and found 27 different islands (Table S1) using IslandViewer 4 [20]. Parts of these islands were found to be present in LAC-4 as well. A large majority of these islands did not contain antibiotic resistance genes and only three of these were identified as resistance islands (Table S1). However, a careful analysis of the identified sequences showed that only two of these three contained antibiotic resistance genes. One island is GI1 (Table 2 and Figure S1a), and the other is ~9 kb (2,458,526-2,467,319 bp) in length, inserted in a gene encoding a hypothetical protein (IX87_RS12440). It harbors two aminoglycoside resistance genes (described below in the section for aminoglycosides) and a β-lactamase gene (Figure 3a) and is absent in LAC-4. The presence of this island has been previously reported in multidrug-resistant *A. baumannii* isolates AbH120-A2 from Spain [21] and AF-401 from Mexico [22] (Figure 3b). This island has also been shown to be present on a 65 kb IncN plasmid in *Klebsiella pneumoniae* KP1050 [23]. It has been suggested that *K. pneumoniae* KP1050 has acquired this from *A. baumannii* [23] (Figure 3b). In our study, the insertion sequence in this island was identified as that belonging to the IS*6* family by IslandViewer (Figure 3a), however since IS*26* is the most commonly studied member of the IS*6* family [24], the insertion sequence in this island may very well be IS*26*.

ISfinder [25] was used to determine the presence of insertion sequences in AB030 and LAC-4. Several insertion sequences, distributed throughout the respective genomes, were found in both strains. AB030 contained 51 insertion sequences and LAC-4 contained 47 IS elements (Figure S2). 

The presence of phage elements was predicted by the PHASTER server [26]. AB030 showed the presence of 14 prophage sequences compared to six in LAC-4. Of the 14 prophage sequences in AB030, five were intact, seven were incomplete, and two were questionable, whereas among the six prophage sequences found in LAC-4, one was intact, four were incomplete, and one was questionable (Table S2). Other than contributing to the larger genome size of AB030, we currently do not know what the significance of additional phage sequences in this strain is.

### 2.4. Antibiotic Susceptibility and Analysis of Antibiotic Resistance Genes

As shown in Table S3, AB030 is an extensive drug-resistant isolate that displays resistance to fluoroquinolones (levofloxacin), carbapenem (imipenem and meropenem), aminoglycosides (amikacin), and tigecycline. AB030, however, displays a much broader resistance profile to different classes of antibiotics than LAC-4 [27].

**Figure 4:**
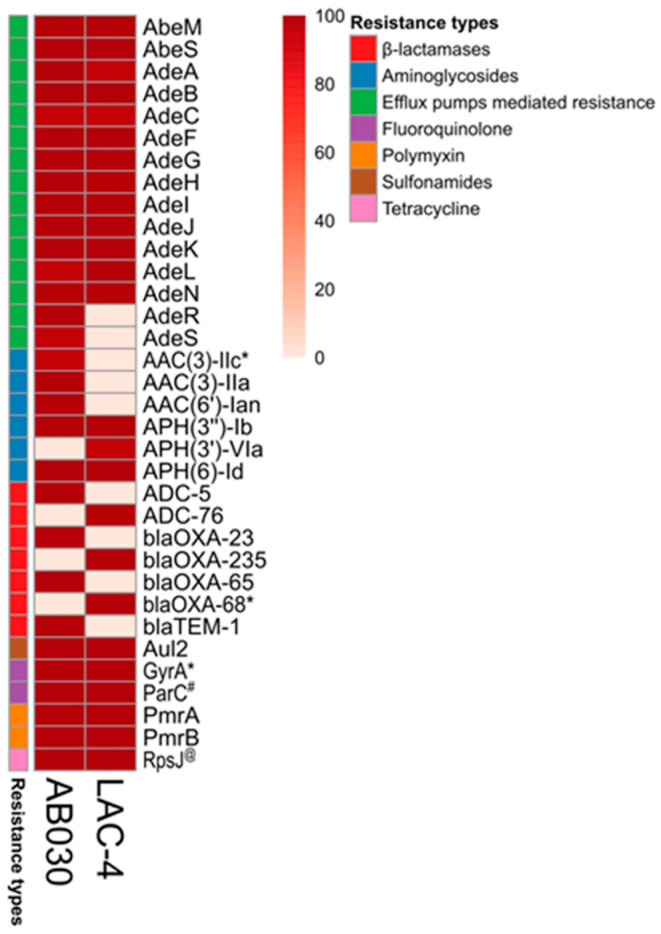
Genome-wide antibiotic resistance genes prediction using CARD database. * A mutation (81S→L) was found in the GyrA of AB030 and LAC-4. Percent identity to the reference gene is indicated in the shades of beige to maroon and the categories of the resistance genes are color-coded. #ParC has 84S→L in AB030, 88E→K in LAC-4, and 467S→G in both AB030 and LAC-4. @ RpsJ contains a 57V→L mutation in AB030.

In order to identify genes potentially responsible for antibiotic resistance in AB030, its chromosome was analyzed along with the chromosome of LAC-4 using the Comprehensive Antibiotic Resistance Database (CARD) [28]. Details on the predicted genes are shown in Figure 4. Genes associated with resistance to different classes of antibiotics in AB030 and LAC-4 are described below.

#### 2.4.1. Fluoroquinolones 

Fluoroquinolone resistance in bacteria is a result of target mutations, such as those in GyrA and ParC [29], in addition to efflux. AB030 contains multiple mutations in both GyrA and ParC: GyrA (^81^S→L) and ParC (^84^S→L, ^467^S→G) (Figure 4). LAC-4, in comparison, contains ^88^E→K and ^467^S→G in ParC. Among different mutations seen in ParC, ^84^S→L and ^88^E→K have been shown to be important in quinolone resistance [30,31], whereas a mutation in GyrA alone generally leads to a modest decrease in susceptibility to quinolones [32]. Nevertheless, a combination of mutations in both ParC and GyrA often leads to clinically significant quinolone resistance [32]. AB030 and LAC-4 display similar susceptibility to ciprofloxacin but AB030 is > eight-fold less susceptible to levofloxacin than LAC-4. It has been shown that susceptibility to different fluoroquinolone antibiotics can differ dramatically in clinical isolates, likely because of differences in the structure of the drug [33]. Susceptibility to ciprofloxacin and levofloxacin also varies depending on the number of mutations in quinolone resistance-determining regions (QRDRs) of *gyr* and *par* genes. For example, *E. coli* and *Klebsiella* spp. mutations in ^83^S and ^87^D together have been shown to be associated with levofloxacin resistance while mutation in ^83^S alone has been associated with ciprofloxacin resistance [34]. It remains to be seen if differences in the mutations found in *parC* of AB030 and LAC-4 contribute to the lower susceptibility of AB030 to levofloxacin compared to LAC-4. 

#### 2.4.2. β-Lactams

AB030 is resistant to carbapenems: meropenem and imipenem [35]. Resistance to carbapenems is particularly concerning as these are considered the “last-resort” antibiotics for treatment of *A. baumannii* infections [36]. AB030’s resistance to carbapenems can be explained by the presence of *bla*_OXA-65_ and two copies of *bla*_OXA-23_ (IX87_RS16500 and IX87_RS21460) genes (Figure 4). These genes, absent in LAC-4, have been shown to confer resistance to carbapenems, specifically, meropenem and imipenem [37]. Interestingly, AB030 contains two copies of *bla*_OXA-23_. *bla*_OXA-23_ can be plasmid-borne or chromosomal and has been reported in both clinical and environmental isolates of *A. baumannii* [38,39]. In AB030, both copies of *bla*_OXA-23_ are flanked by ISAba1 insertion sequences. AB030 also contains *bla*_OXA-65_ while LAC-4 contains *bla*_OXA-68_. The genetic locations of these genes were identical in both isolates (Figure S3a). These two genes are classified as *bla*_OXA-51_^-^like and their products have a greater affinity for imipenem than meropenem [40]. Identity of each of the two genes was confirmed by sequence alignment with published sequences of *bla*_OXA-65_ and *bla*_OXA-68_ [41] (Figure S3b).

CARD analysis also showed the presence of ADC beta-lactamases in both AB030 and LAC-4 (Figure S4). ADC or *Acinetobacter*-derived cephalosporins are active against extended-spectrum cephalosporins but not against carbapenems [42]. AB030 contains *bla*_ADC-5_ and LAC-4 contains *bla*_ADC-76_. *bla*_ADC-5_ has previously been reported in *Acinetobacter pittii* [43]. On the other hand, *bla*_ADC-76_, found in LAC-4, is rare and has only been reported in one other clinical isolate of *A. baumannii* [44].

#### 2.4.3. Aminoglycosides

Aminoglycoside resistance in *A. baumannii* is widely reported [45]. High levels of resistance is often attributed to the modification of aminoglycosides by acetyltransferases (AAC) [46]. AB030 contains several aminoglycoside resistance genes (Figure 4), including *aac* genes located on a mobile element (also described above in the section for beta-lactams) (Figure 3a,b). This mobile element, reported in two other multidrug-resistant *A. baumannii* strains, AbH12O-A2 [21] and AF-401 [22] (Figure 3b), contains *aac(3)-IIa* and *aac(6’)-Ian* that flank the beta-lactamase *blaTEM-1. aac(3)-IIa* confers resistance to gentamicin and has been reported in relatively few isolates of *A. baumannii* [47]. *aac(6’)-Ian*, on the other hand, is a novel amikacin resistance determinant reported recently in *Serratia marcescens* [48]. 

Both AB030 and LAC-4 also contain aminoglycoside phosphotransferase (APH)-encoding genes. Of these, APH(3”)-Ib and APH(6)-Id were found in both isolates and their transfer is potentially mediated by an ISAba1 element encoded upstream of *APH*(3”)-Ib (Figure S5).

#### 2.4.4. Tigecycline 

**Figure 5:**
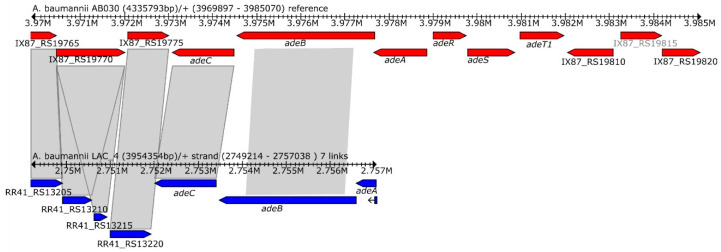
Genetic organization of AdeABC RND-efflux system and two-component system, AdeRS in AB030 (red) and LAC-4 (blue). The adeRS operon that encodes the regulator of the AdeABC system is missing in the LAC-4 genome.

Tigecycline is an important antibiotic for the treatment of MDR *A. baumannii* infections [49]. AB030’s resistance to tigecycline (Table S3) indicates that treatment options for isolates such as AB030 are extremely limited. Lower susceptibility of AB030 to tigecycline can perhaps be explained via a point mutation ^57^V→L in RpsJ (30S ribosomal protein S10) (Figure 4). ^57^V→L in RpsJ mutation is shown to be quite frequent in *A. baumannii* isolates when exposed to tetracycline [50]. Further, expression of the AdeABC pump is an important determinant of tigecycline resistance in *A. baumannii* [51]. This pump is overexpressed in AB030 [35], which may also at least be partially responsible for AB030’s resistance to tigecycline. Expression of the AdeABC system is activated by a two-component system AdeRS [52]. LAC-4 is missing the *adeRS* operon in its entirety (Figure 4 and Figure 5). We did not analyze the expression of AdeABC in LAC-4 and it is not clear what impact the absence of the *adeRS* operon has on the expression of the AdeABC pump. 

### 2.5. Virulence of A. baumannii Strains in Mouse Infection Model and Analysis of Virulence Genes

**Figure 6:**
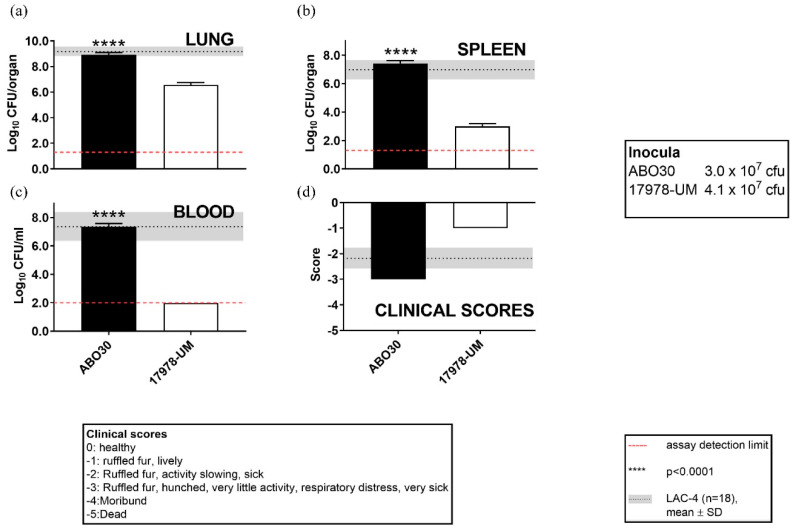
Bacterial burdens in lung (**a**), spleen (**b**), and blood (**c**). Mice (*n* = 5/group) were intranasally inoculated with AB030 and the type strain ATCC17978. The clinical signs were scored as indicated (**d**). The mice were sacrificed at 24 hours and their blood, lung, and spleen collected for quantitative bacteriology. The dotted lines in red indicate the limit of detection. Data for AB030 and ATCC17978 were compared with pooled data from 18 mice for LAC-4 (shown as gray horizontal bars) from four independent experiments. The bacterial burden data were analyzed by one-way ANOVA followed by post hoc Dunnett’s multiple comparison test.

Genes associated with virulence factors in AB030 and LAC-4 are summarized in Table S3. As is evident from the table, AB030 harbors more virulence-associated genes compared to LAC-4. In order to test the virulence of AB030, the intranasal mice infection model was used [27]. Dissemination of AB030 to lung, spleen, and blood was assessed after 24 h of infection as a determinant of pathogenicity. *A. baumannii* ATCC17978 was used as the control. As shown in Figure 6a–c, AB030 was as efficient as LAC-4 in colonizing lungs, spleen, and blood of the infected mice. However, the clinical outcome of mice infected with AB030 was slightly poorer than that for LAC-4, indicating that AB030 may be more virulent than LAC-4 (Figure 6d). These data show that in addition to being hyper-resistant to antibiotics, AB030 is also hypervirulent. 

Following the confirmation of the hypervirulent phenotype, AB030 was tested for specific virulence-associated phenotypes, such as biofilm formation, motility, and catalase activity. These phenotypes were chosen based on the underlying genetic differences among these strains as listed in Table S4. 

**Figure 7:**
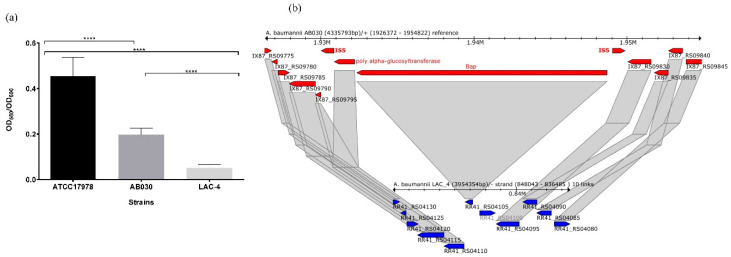
Biofilm formation by AB030 in comparison to LAC-4 and ATCC17978 (**a**). Organization of bap (biofilm-associated protein-encoding gene) in AB030 (red) and LAC-4 (blue) (**b**). There is insertion of an IS5 family transposases before the start codon of the bap gene in AB030 which may disrupt its transcription. Statistical analysis was carried out using one-way ANOVA with Tukey’s multiple comparison test. **** denotes *p* < 0.0001.

Biofilm formation is an important virulence factor in pathogenic bacteria [53]. It can result in persistent, difficult-to-eradicate infections. We observed that AB030 was more efficient in forming biofilm compared to LAC-4 (Figure 7a) but less efficient than the type strain ATCC17978. Bap protein has been shown to be essential for biofilm formation in *A. baumannii* and is involved in biotic surface adherence [54]. *bapA* in AB030 is flanked by the ISAba27 insertion element. In *Staphylococcus aureus*, SarA protein has been shown to activate the expression of *bap* by binding to the promoter region [55]. While no such activator of *bapA* expression has been shown in *A. baumannii*, it is possible that the insertion in the promoter region of *bapA* in AB030 affects its expression and is likely to impact the biofilm formation (Figure 7b). Another factor suggested to be modulating biofilm in *A. baumannii* is the availability of iron for the cell. An increased biofilm formation has been observed in *A. baumannii* ATCC19606 in the absence of iron [56], similar observations have been reported in *Pseudomonas aeruginosa* [57]. However, a recent study failed to establish a clear link between iron availability and biofilm formation in veterinary and clinical isolates of *A. baumannii* [58]. Nevertheless, we did not find any obvious differences in the set of iron uptake genes in AB030 and LAC-4 (Supplementary Table S3) which may result in higher biofilm formation. LAC-4 was found to form little or no biofilm (Figure 7a). LAC-4 contains cryptic *bap* (Figure 7b) and therefore the factors leading to its inability to produce biofilms are perhaps similar to those of AB030. However, in addition to the disrupted *bap*, LAC-4 lacks *abaI* (LuxI synthase) and *abaR* (LuxR receptor) genes (Supplementary Table S3), both of which are part of the quorum sensing system in *A. baumannii* [59] and mutational inactivation of *abaI* has been shown to result in up to 40% reduction in biofilm formation in *A. baumannii* strains [60].

**Figure 8:**
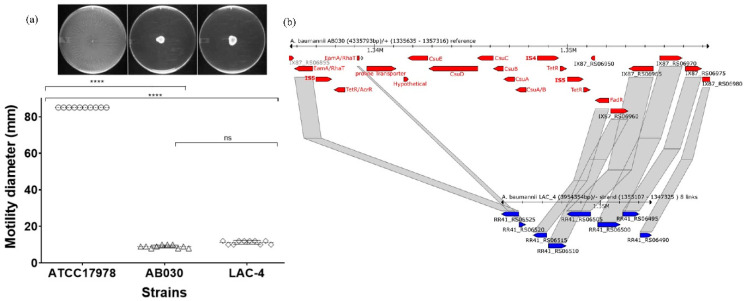
Motility of AB030 compared to that of LAC-4 and ATCC17978 (**a**). Organization of the csu operon in AB030 (red) and LAC-4 (blue) (**b**). Statistical analysis was carried out using the one-way (for motility assays) and two-way (for biofilm assays) analysis of variance (ANOVA) (****, *p* < 0.0001).

Motility is also considered an important virulence factor in bacteria [61]. Since *A. baumannii* lacks any flagellar structure, its surface motility (twitching) is mediated through pili [61,62]. The pili-mediated motility requires surface attachments which are provided by the proteins encoded by the *csuABCDE* operon [56]. AB030 and LAC-4 both did not display any motility on the agarose medium (Figure 8a). An analysis of the *csu* operon revealed that even though AB030 contains the intact *csu* operon (organized in identical fashion as in ATCC17978), it has insertions of ISAba1 and ISAba27, which are not present in ATCC17978, immediately upstream of the operon (Figure 8b). A two-component system CheA/Y has been shown to upregulate the expression of the *csu* operon [63] and the presence of the insertion elements upstream of the *csu* operon may impact its expression as mediated by the CheA/Y system. This may explain lack of motility observed in AB030 (Figure 8a). LAC-4, on the other hand, lacks the entire *csu* operon (Figure 8b), which may explain its lack of motility on agarose medium (Figure 8a). 

The presence of catalase has been linked to bacterial pathogens’ resistance to host immune factors that employ reactive oxygen species such as hydrogen peroxide [64]. Both AB030 and LAC-4 displayed an increased catalase activity compared to ATCC17978, with AB030 showing the higher activity (Figure S6). In *A. baumannii*, KatG and KatE are the predominant catalases [65]. In addition, KatA and KatX have been described in *Acinetobacter* but their role in H_2_O_2_ resistance is not clear [65]. AB030 also contained *katA*, in addition to *katG* and *katE* (Table S4). This may explain slightly higher catalase activity observed in AB030, which may partially aid its hypervirulence. 

## 3. Materials and Methods

### 3.1. Bacterial Strains and Growth Conditions

Two clinical isolates of *A. baumannii*, AB030 (NZ_CP009257.1) [10] and LAC-4 (NZ_CP007712.1) [11], were used in this study. Annotation of both these strains has been carried out automatically with the National Center for Biotechnology Information (NCBI) Prokaryotic Genome Annotation Pipeline (PGAP). ATCC17978 was used as the control strain. Bacterial strains were grown in LB broth (Becton, Dickenson and Company, MD, USA) at 37 °C with 250 rpm shaking unless otherwise stated.

### 3.2. Genome Alignment and Functional Assignation Analysis

Genomic rearrangements between AB030 and LAC-4 sequences were visualized by performing progressive Mauve aligner [66] with the Geneious software (version 11.0) [67] utilizing default parameters. GView server (https://server.gview.ca/) at 95% identity was used to compare the genomes of these two isolates. Comparative genome visualization was performed with CloVR version 1.0-RC9 [68]. All the annotated protein-coding sequences (CDS) from the above genomes were cross-referenced for orthologous sequences in the Clusters of Orthologous Groups (COG) database available in the NCBI CDD web search tool [69].

### 3.3. Genomic Islands, Phage Sequences, and Insertion Sequences

Insertion sequences in the two genomes were identified using ISfinder, a web-based tool which searches for insertion sequences in provided nucleotide sequences based on the sequences in their database [25]. The insertion sequences were considered a positive “hit” when their E-value was recorded below 0.01. Phage elements were identified using the PHASTER database [26]. Pathogenic islands were identified using the IslandViewer 4 database [20].

### 3.4. Antibiotic Susceptibility and Prediction of Antibiotic Resistance Genes

Antibiotic susceptibility for AB030 and LAC-4 have been published previously [11,35]. Antibiotic resistance genes were predicted using the Comprehensive Antibiotic Resistance Database (CARD) with “strict” and “perfect” parameters [28] using the genomes of AB030 (NZ_CP009257.1) and LAC-4 (NZ_CP007712.1). The presence/absence of predicted antibiotic resistance genes in three genomes were confirmed by searching the nucleotide sequences in the respective genomes using the BLASTn tool available in the NCBI server. 

### 3.5. Identification of Virulence Genes

Genes encoding virulence factors were identified using the virulence factor (VF) analyzer online tool [70]. Presence and absence of virulence genes in three genomes were confirmed by searching nucleotide sequences in respective genomes using the BLASTn tool available in the NCBI server.

### 3.6. Intranasal A. baumannii Infection Assay

Adult (20–22 weeks old) pathogen-free female BALB/c mice were purchased from Charles River Laboratories (St. Constant, QC, Canada). The mice were maintained and used in accordance with the recommendations of the Canadian Council on Animal Care Guide to the Care and Use of Experimental Animals. All experimental protocols and procedures were approved by the institutional animal care committee, Human Health Therapeutics, National Research Council Canada (Animal User Protocol #2016.11). 

Intranasal mice infection assays in female BALB/c mice were carried out as previously described [27]. Briefly, mice were anesthetized by intraperitoneal (i.p.) injection of 4% isoflurane with medical O_2_ and then inoculated intranasally with 5 × 10^7^ CFU *A. baumannii* ATCC17978 and AB030 from fresh inocula in 50 μL of saline. Actual inocula in each experiment were determined by plating 10-fold serial dilutions on brain heart infusion agar plates. The clinical appearance of the mice was monitored and scored as described previously [27]. Groups of five infected mice were sacrificed 24 h post-inoculation (hpi). Quantitative bacteriology was carried out from lungs, spleens, and blood after their removal aseptically. Data obtained for ATCC17978 and AB030 were compared with data for LAC-4 obtained using a total of 18 mice (inoculum size of 2–6 × 10^7^) from four experiments carried out on separate days from those for ATCC17978 and AB030. 

### 3.7. Biofilm Assay

Biofilm formation was assayed based on a previously described method [71] with slight modifications. Briefly, bacterial strains were grown in 3 mL cultures overnight at 37 °C with 250 rpm shaking in LB broth (Becton, Dickenson and Company, MD, USA). The overnight cultures were then diluted 1:100 (v/v) in the same medium and 100 µL aliquots were distributed in individual wells of a 96-well round-bottom microplate (Sarstedt, Montréal, QC, Canada). The 96-well plates were then incubated at 37 °C in a static incubator for 48 h to allow the biofilm formation following which A_600_ was measured to determine the growth of the cultures using a SpectraMax M2 (Molecular Devices, San Jose, CA, USA). The culture medium and planktonic cells were removed by inverting the plates and washing wells twice with dH_2_O. One hundred µL of 0.5% (w/v) crystal violet was then added to each well and plates incubated for 20 min at room temperature before removing the crystal violet and repeating the washing as described above. Finally, to quantify the biofilm, 100 µL of acetone:ethanol (2:1) mixture was added to each well and incubated for 20 min and A_580_ determined. Biofilm formation was calculated by normalizing the A_580_ value with the A_600_ value for each well, plotted and statistically analyzed using GraphPad Prism v6.07 (La Jolla, CA, USA). The assays were carried out in biological duplicates with 16 technical replicates for each biological replicate. 

### 3.8. Motility Assay

The surface motility was assayed based on a previously described method [62] using 0.3% agarose plates for each strain with slight modifications. Briefly, 3 µL of the overnight cultures of each strain were stab inoculated onto the center of freshly prepared 0.3% agarose plates and incubated for 18 h at 37 °C. The diameter of the region showing bacterial growth was measured after the incubation period and representative plates were photographed. Results were plotted and statistically analyzed using GraphPad Prism v6.07 (La Jolla, CA, USA). The assays were carried out with 10 biological replicates. 

### 3.9. Catalase Activity Assay

Bacterial strains were grown overnight at 37 °C with 250 rpm shaking in LB broth into lag phase prior to the assay in a total volume of 5 mL in a 50 mL conical tube (Sarstedt, Montréal, QC, Canada). Absorbance at 600 nm was measured prior to the assay for the purpose of normalization of the catalase activity based on the growth of each strain. The catalase activity assay was carried out using a Gilson oxygraph equipped with a Clark electrode as described previously [72]. The assay was carried out in biological duplicates and the average values with the standard deviations were plotted and statistically analyzed using GraphPad Prism v6.07 (La Jolla, CA, USA).

## 4. Conclusions

In summary, this study shows that genome plasticity and accumulation of resistance genes via insertion elements contribute to the extreme drug resistance phenotype of *A. baumannii* AB030, a member of the newly emerging lineage of *A. baumannii*. Genomic and phenotypic comparison of AB030 with LAC-4 shows that AB030 has accumulated a number of genes that may contribute to its hyper-resistance and hypervirulence. Our data also show that biofilm formation on an abiotic surface and motility on agarose medium did not impact the virulence of AB030 and LAC-4 in mice models. This underscores the importance of other factors in the hypervirulence of AB030. Even though this work is limited to only one isolate of ST758 lineage and currently it is not clear if the other members of this lineage share the same phenotype as AB030, it highlights the importance of characterization of members of emerging lineages of *A. baumannii* for their virulence and antibiotic resistance. 

## Figures and Tables

**Table 1:** Genome features of AB030 and LAC-4.

Feature	AB030	LAC-4
Number of base pairs	4,335,793	3,954,354
G+C content	39%	39%
CDS (total)	4200	3745
Genes (total)	4296	3839
Genes (RNA)	96	94

**Table 2:** A comparison of genomic islands (GIs) found in *A. baumannii* LAC-4 with those in *A. baumannii* AB030. Alignment of GIs is shown in Supplementary Figure S1.

Genomic Islands in LAC-4	Location in LAC-4 Genome	Island Length (bp)	AB030	
			Coverage	Sequence Identity
GI1 (Suppl. Figure S1a)	120,830–133,655	12825	100	100%
GI2 (Suppl. Figure S1b)	547,857–581,621	33764	-	-
GI3 (Suppl. Figure S1c)	788,377–800,975	12598	61%	99%
GI4 (Suppl. Figure S1d)	940,403–951,920	11517	78%	99%
GI5 (Suppl. Figure S1e)	1,229,550–1,257,942	28392	74%	96%
GI6 (Suppl. Figure S1f)	1,571,326–1,611,225	39899	62%	94%
GI7 (Suppl. Figure S1g)	1,730,338–1,741,047	10709	59%	100%
GI8 (Suppl. Figure S1h)	1,941,480–1,993,076	51596	-	-
GI9 (Suppl. Figure S1i)	2,963,891–2,993,411	29520	48%	94%
GI10 (Suppl. Figure S1j)	3,028,724–3,058,412	29688	48%	96%
GI11 (Suppl. Figure S1k)	3,367,720–3,386,189	18469	5%	80%
GI12 (Suppl. Figure S1l)	3,852,499–3,882,676	30177	58%	100%

## Supplementary Materials

The following are available online at https://www.mdpi.com/2079-6382/9/6/328/s1, Figure S1. Alignment of genomic islands (GIs) described in *A. baumannii* LAC-4 [11] with those in *A. baumannii* AB030. Figure S2. Insertion Sequence (IS regions) in AB030 and LAC-4 genomes as predicted by ISfinder. Presence of the IS region is indicated by red and the absence by blue. Figure S3. Presence of *bla*_OXA_ in *A. baumannii* AB030 and LAC-4 and alignment of each of the three genes with published sequences of *bla*_OXA-65_ and *bla*_OXA-68_ [41]. Figure S4. Presence of *bla*_ADC_ in *A. baumannii* AB030 and LAC-4. Figure S5. Presence of aminoglycoside phosphotransferase (APH)-encoding genes *APH*(3″)-Ib and *APH*(6)-Id in *A. baumannii* AB030 and LAC-4. Figure S6. Catalase activity of AB030 and LAC-4, in comparison to ATCC 17978. Table S1. Predicted genomic islands present in AB030 as identified by Islandviewer. Table S2. Predictions for the presence of phage elements in the genomes of AB030 and LAC-4. Table S3. Antibiotic susceptibility (μg/mL) assay of AB030 and LAC-4. Table S4. Presence of virulence genes in AB030 and LAC-4.
