# Supplementary material for: Characterization of Extremely Drug-Resistant and Hypervirulent Acinetobacter baumannii AB030
Source: Antibiotics (Basel). 2020 Jun 17;9(6):328. doi: 10.3390/antibiotics9060328 (PMC7345994; doi:10.3390/antibiotics9060328)
Supplement: Supplementary file 1 [file antibiotics-09-00328-s001.pdf]

Supplementary Figures and Tables

Fig. S1. Alignment of Genomic Islands (GIs) (marked by the region between green arrows) described in *A. baumannii* LAC-4 [11] with those in *A. baumannii* AB030. Twelve different GIs have been described *A. baumannii* LAC-4: GI1 (a), GI2 (b), GI3 (c), GI4 (d), GI5 (e), GI6 (f), GI7 (g), GI8 (h), GI9 (i), GI10 (j), GI11 (k), and GI12 (l). Genes of interest are listed in red font, genes that are similar in both strains are listed in black font, and genes that are found in only one of the two strains are listed in gray font.

Fig. S2. Insertion Sequence (IS regions) in AB030 and LAC-4 genomes as predicted by ISfinder. Presence of the IS region is indicated by red and the absence by blue.

Fig. S3. Presence of bla_{OXA} in *A. baumannii* AB030 and LAC-4 (a) and alignment of each of the three genes with published sequences of $bla_{\mathrm{OXA\text{-}65}}$ and $bla_{\mathrm{OXA\text{-}68}}$ [39] (b).

Fig. S4. Presence of bla_{ADC} in *A. baumannii* AB030 and LAC-4.

Fig. S5. Presence of aminoglycoside phosphotransferase (APH) encoding genes, *APH*(3”)-Ib and *APH*(6)-Id in *A. baumannii* AB030 and LAC-4.

Fig. S6. Catalase activity of AB030 and LAC-4, in comparison to ATCC 17978. Catalase activity toward H_2O_2 in overnight cultures was determined using a Clark oxygen electrode connected to a Gilson oxygraph. Values were normalized to the dry weight of bacteria. Results represent data from two independent experiments. Statistical analysis was carried out using the two-way analysis of variance (ANOVA).

Fig. S1a

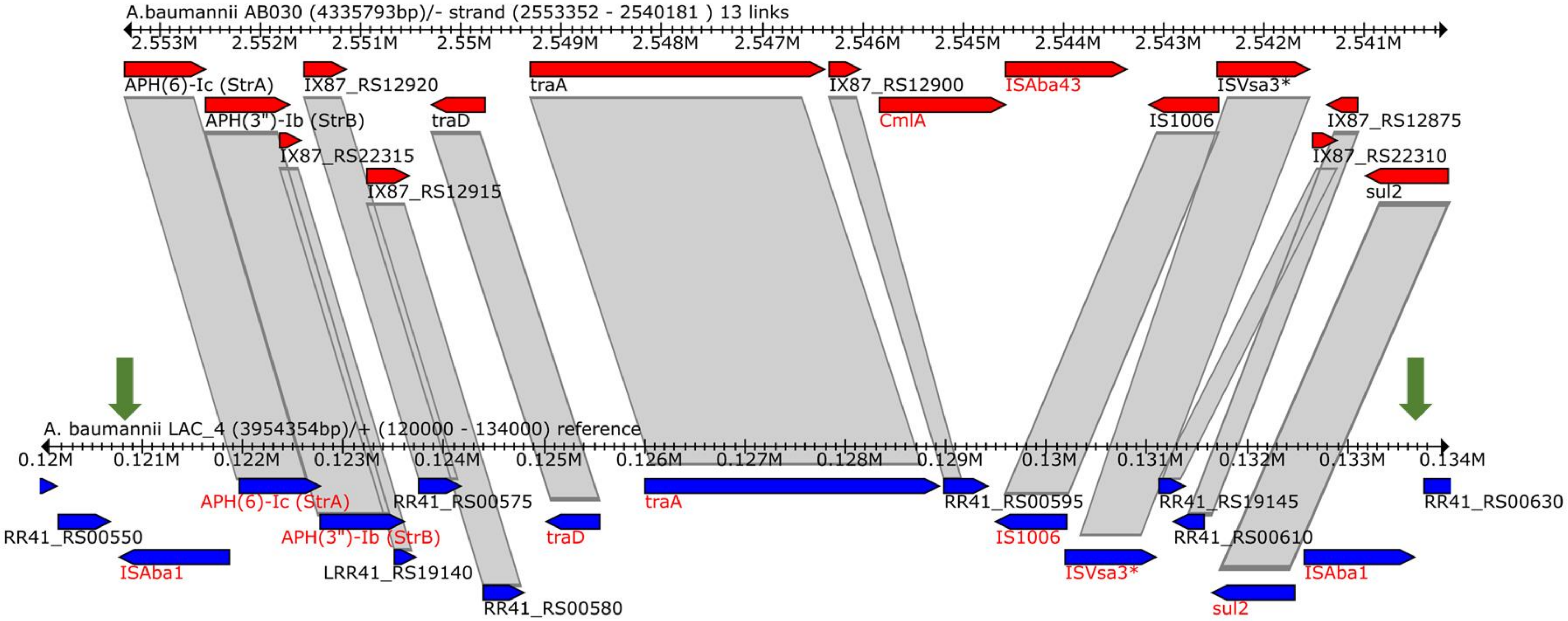
A.baumannii AB030 (4335793bp)/- strand (2553352 - 2540181) 13 links
2.553M
2.552M
2.551M
2.55M
2.549M
2.548M
2.547M
2.546M
2.545M
2.544M
2.543M
2.542M
2.541M
APH(6)-Ic (StrA)
IX87_RS12920
traA
IX87_RS12900
ISAba43
ISVsa3*
APH(3")-Ib (StrB)
traD
CmlA
IS1006
IX87_RS12875
IX87_RS22315
IX87_RS22310
IX87_RS12915
sul2
A. baumannii LAC_4 (3954354bp)/+ (120000 - 134000) reference
0.12M
0.121M
0.122M
0.123M
0.124M
0.125M
0.126M
0.127M
0.128M
0.129M
0.13M
0.131M
0.132M
0.133M
0.134M
APH(6)-Ic (StrA)
RR41_RS00575
traA
RR41_RS00595
RR41_RS19145
RR41_RS00630
RR41_RS00550
APH(3")-Ib (StrB)
traD
IS1006
RR41_RS00610
ISAba1
LRR41_RS19140
ISVsa3*
ISAba1
RR41_RS00580
sul2

Fig. S1b

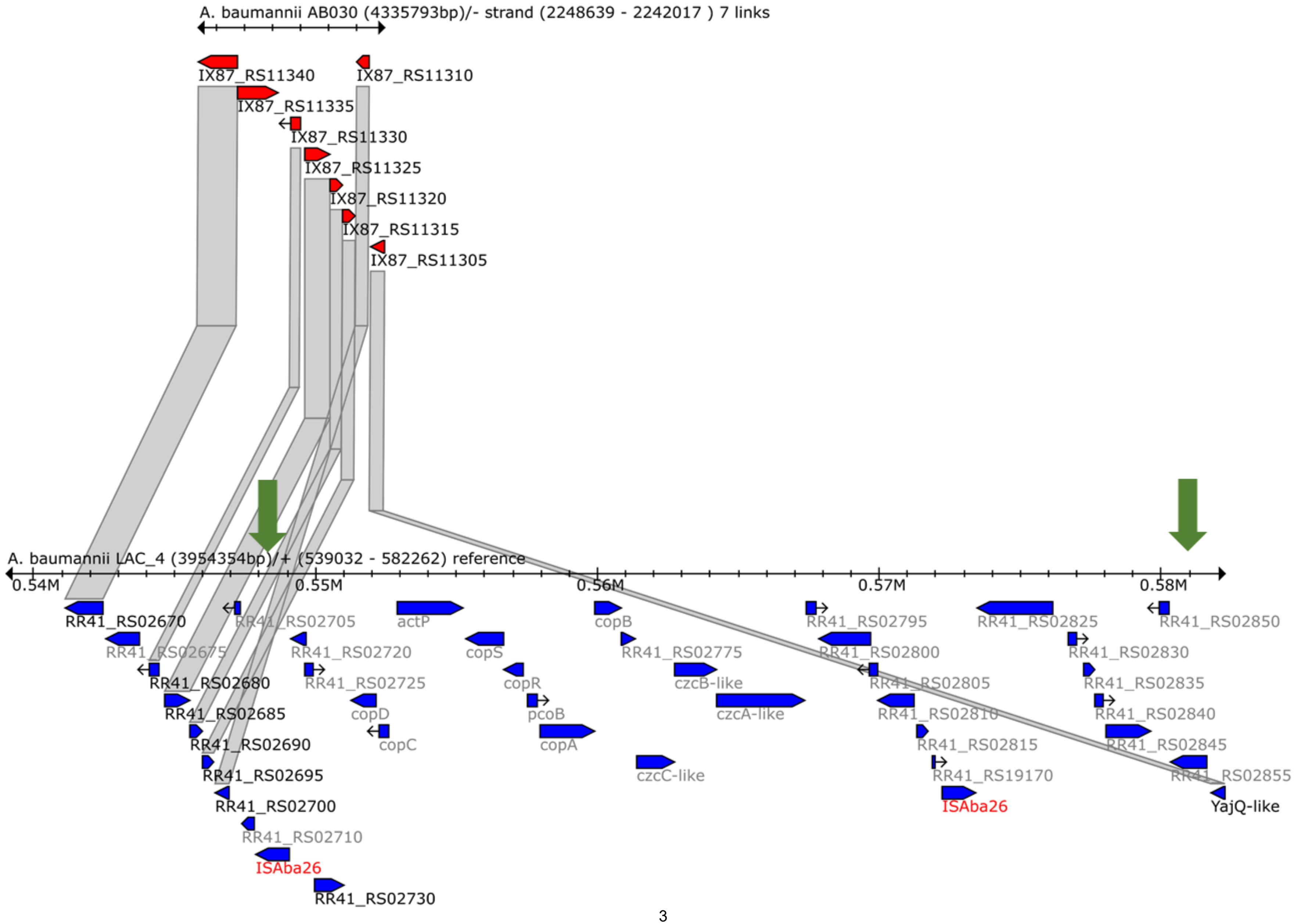
A. baumannii AB030 (4335793bp)/- strand (2248639 - 2242017) 7 links
IX87_RS11340
IX87_RS11310
IX87_RS11335
IX87_RS11330
IX87_RS11325
IX87_RS11320
IX87_RS11315
IX87_RS11305
A. baumannii LAC_4 (3954354bp)/+ (539032 - 582262) reference
0.54M
0.55M
0.56M
0.57M
0.58M
RR41_RS02670
RR41_RS02705
actP
copB
RR41_RS02795
RR41_RS02825
RR41_RS02850
RR41_RS02675
RR41_RS02720
copS
RR41_RS02775
RR41_RS02800
RR41_RS02830
RR41_RS02680
RR41_RS02725
copR
czcB-like
RR41_RS02805
RR41_RS02835
RR41_RS02685
copD
pcoB
czcA-like
RR41_RS02810
RR41_RS02840
RR41_RS02690
copC
copA
RR41_RS02815
RR41_RS02845
RR41_RS02695
czcC-like
RR41_RS19170
RR41_RS02855
RR41_RS02700
ISAba26
YajQ-like
RR41_RS02710
ISAba26
RR41_RS02730

Fig. S1c

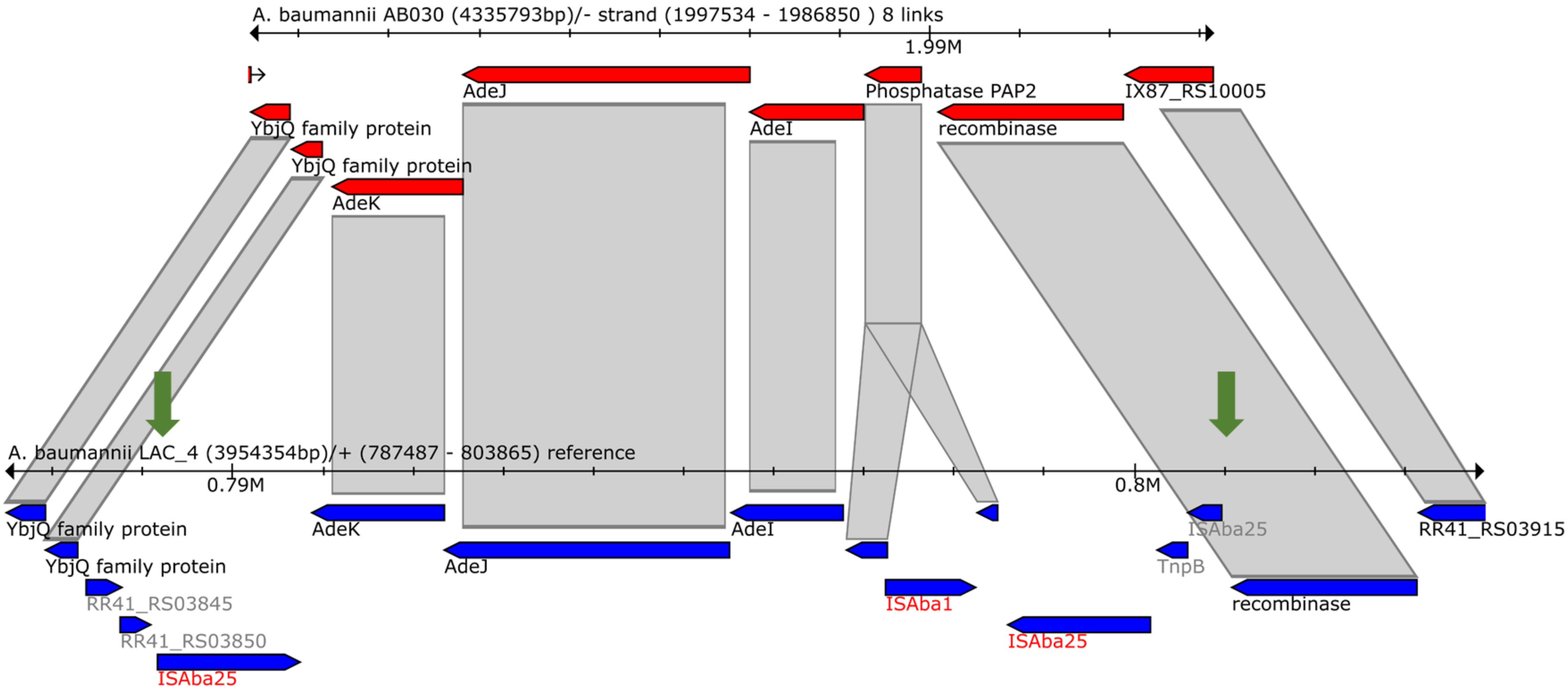
A. baumannii AB030 (4335793bp)/- strand (1997534 - 1986850) 8 links
1.99M
AdeJ
Phosphatase PAP2
IX87_RS10005
YbjQ family protein
AdeI
recombinase
YbjQ family protein
AdeK
A. baumannii LAC_4 (3954354bp)/+ (787487 - 803865) reference
0.79M
0.8M
YbjQ family protein
AdeK
AdeI
ISAba25
RR41_RS03915
YbjQ family protein
AdeJ
TnpB
RR41_RS03845
ISAba1
recombinase
RR41_RS03850
ISAba25
ISAba25

Fig. S1d

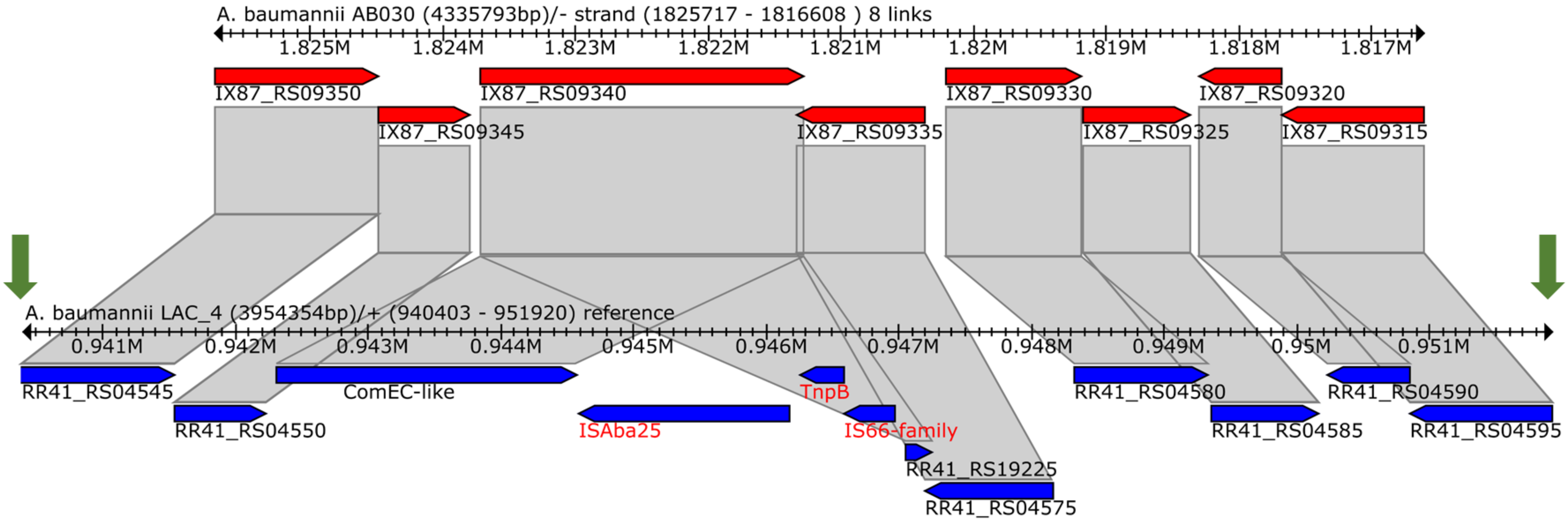
A. baumannii AB030 (4335793bp)/- strand (1825717 - 1816608) 8 links
1.825M
1.824M
1.823M
1.822M
1.821M
1.82M
1.819M
1.818M
1.817M
IX87_RS09350
IX87_RS09340
IX87_RS09330
IX87_RS09320
IX87_RS09345
IX87_RS09335
IX87_RS09325
IX87_RS09315
A. baumannii LAC_4 (3954354bp)/+ (940403 - 951920) reference
0.941M
0.942M
0.943M
0.944M
0.945M
0.946M
0.947M
0.948M
0.949M
0.95M
0.951M
RR41_RS04545
ComEC-like
TnpB
RR41_RS04580
RR41_RS04590
RR41_RS04550
ISAba25
IS66-family
RR41_RS04585
RR41_RS04595
RR41_RS19225
RR41_RS04575

Fig. S1e

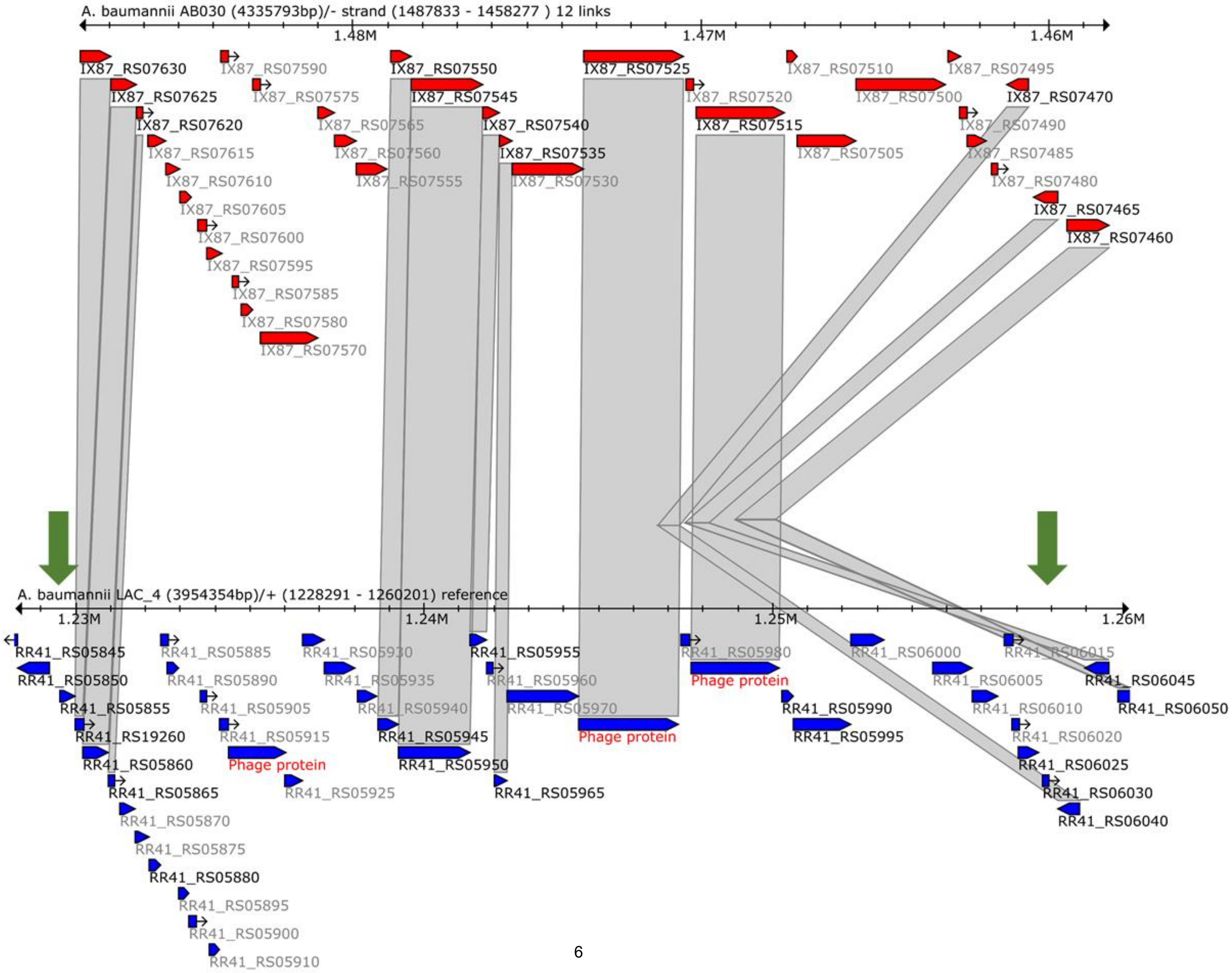
A. baumannii AB030 (4335793bp)/- strand (1487833 - 1458277) 12 links
1.48M
1.47M
1.46M
IX87_RS07630
IX87_RS07590
IX87_RS07550
IX87_RS07525
IX87_RS07510
IX87_RS07495
IX87_RS07625
IX87_RS07575
IX87_RS07545
IX87_RS07520
IX87_RS07500
IX87_RS07470
IX87_RS07620
IX87_RS07565
IX87_RS07540
IX87_RS07515
IX87_RS07490
IX87_RS07615
IX87_RS07560
IX87_RS07535
IX87_RS07505
IX87_RS07485
IX87_RS07610
IX87_RS07555
IX87_RS07530
IX87_RS07480
IX87_RS07605
IX87_RS07465
IX87_RS07600
IX87_RS07460
IX87_RS07595
IX87_RS07585
IX87_RS07580
IX87_RS07570
A. baumannii LAC_4 (3954354bp)/+ (1228291 - 1260201) reference
1.23M
1.24M
1.25M
1.26M
RR41_RS05845
RR41_RS05885
RR41_RS05930
RR41_RS05955
RR41_RS05980
RR41_RS06000
RR41_RS06015
RR41_RS05850
RR41_RS05890
RR41_RS05935
RR41_RS05960
Phage protein
RR41_RS06005
RR41_RS06045
RR41_RS05855
RR41_RS05905
RR41_RS05940
RR41_RS05970
RR41_RS05990
RR41_RS06010
RR41_RS06050
RR41_RS19260
RR41_RS05915
RR41_RS05945
Phage protein
RR41_RS05995
RR41_RS06020
RR41_RS05860
Phage protein
RR41_RS05950
RR41_RS06025
RR41_RS05865
RR41_RS05925
RR41_RS05965
RR41_RS06030
RR41_RS05870
RR41_RS06040
RR41_RS05875
RR41_RS05880
RR41_RS05895
RR41_RS05900
RR41_RS05910

Fig. S1f

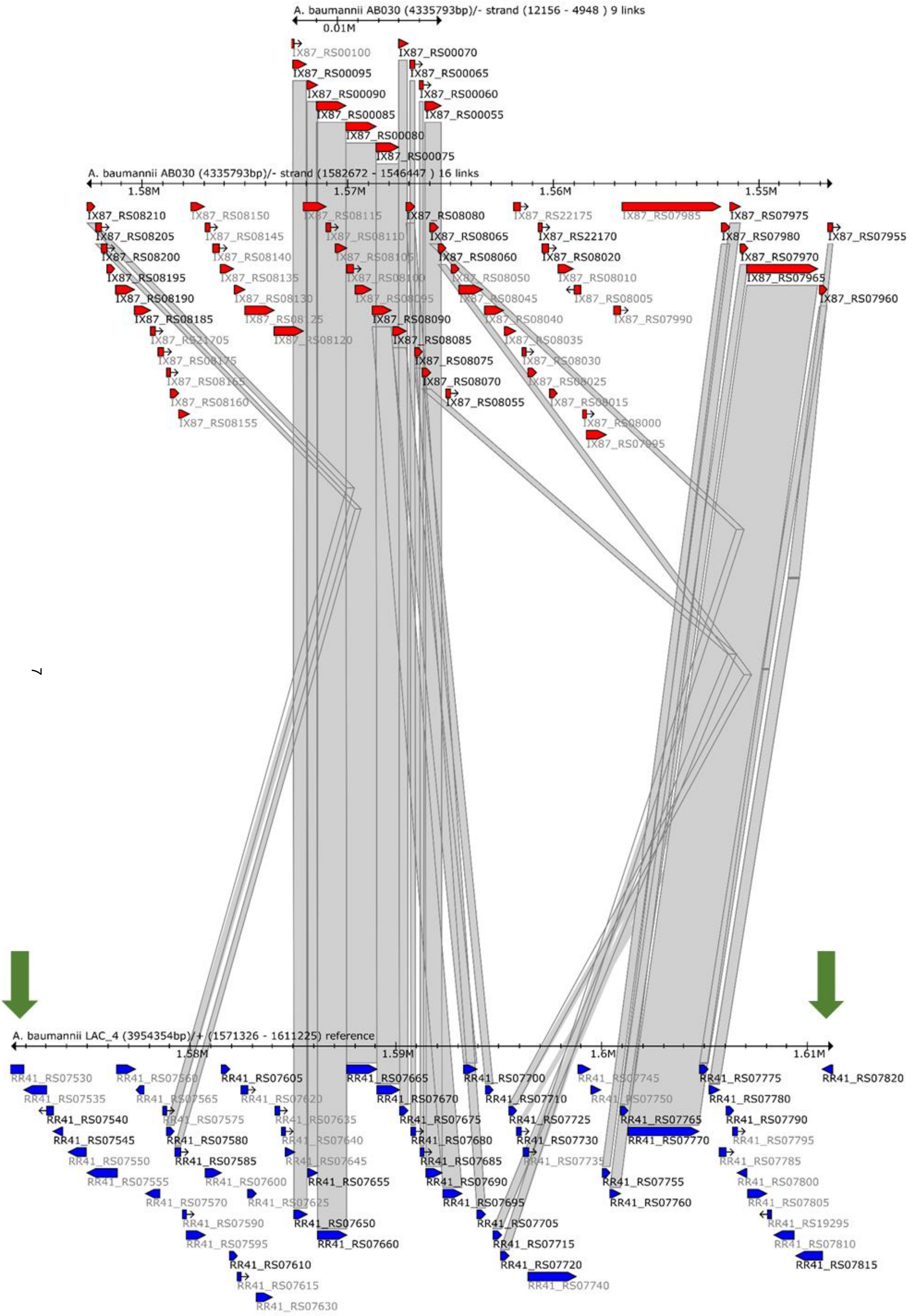
A. baumannii AB030 (4335793bp)/- strand (12156 - 4948) 9 links
0.01M
IX87_RS00100
IX87_RS00095
IX87_RS00090
IX87_RS00085
IX87_RS00080
IX87_RS00075
IX87_RS00070
IX87_RS00065
IX87_RS00060
IX87_RS00055
A. baumannii AB030 (4335793bp)/- strand (1582672 - 1546447) 16 links
1.58M
1.57M
1.56M
1.55M
IX87_RS08210
IX87_RS08205
IX87_RS08200
IX87_RS08195
IX87_RS08190
IX87_RS08185
IX87_RS21705
IX87_RS08175
IX87_RS08165
IX87_RS08160
IX87_RS08155
IX87_RS08150
IX87_RS08145
IX87_RS08140
IX87_RS08135
IX87_RS08130
IX87_RS08125
IX87_RS08120
IX87_RS08115
IX87_RS08110
IX87_RS08105
IX87_RS08100
IX87_RS08095
IX87_RS08090
IX87_RS08085
IX87_RS08080
IX87_RS08075
IX87_RS08070
IX87_RS08065
IX87_RS08060
IX87_RS08055
IX87_RS08050
IX87_RS08045
IX87_RS08040
IX87_RS08035
IX87_RS08030
IX87_RS08025
IX87_RS08015
IX87_RS08000
IX87_RS07995
IX87_RS22175
IX87_RS22170
IX87_RS08020
IX87_RS08010
IX87_RS08005
IX87_RS07990
IX87_RS07985
IX87_RS07975
IX87_RS07980
IX87_RS07970
IX87_RS07965
IX87_RS07955
IX87_RS07960
A. baumannii LAC_4 (3954354bp)/+ (1571326 - 1611225) reference
1.58M
1.59M
1.6M
1.61M
RR41_RS07530
RR41_RS07535
RR41_RS07540
RR41_RS07545
RR41_RS07550
RR41_RS07555
RR41_RS07560
RR41_RS07565
RR41_RS07575
RR41_RS07580
RR41_RS07585
RR41_RS07600
RR41_RS07570
RR41_RS07590
RR41_RS07595
RR41_RS07610
RR41_RS07615
RR41_RS07630
RR41_RS07605
RR41_RS07620
RR41_RS07635
RR41_RS07640
RR41_RS07645
RR41_RS07655
RR41_RS07625
RR41_RS07650
RR41_RS07660
RR41_RS07665
RR41_RS07670
RR41_RS07675
RR41_RS07680
RR41_RS07685
RR41_RS07690
RR41_RS07695
RR41_RS07700
RR41_RS07710
RR41_RS07725
RR41_RS07730
RR41_RS07735
RR41_RS07705
RR41_RS07715
RR41_RS07720
RR41_RS07740
RR41_RS07745
RR41_RS07750
RR41_RS07765
RR41_RS07770
RR41_RS07755
RR41_RS07760
RR41_RS07775
RR41_RS07780
RR41_RS07790
RR41_RS07795
RR41_RS07785
RR41_RS07800
RR41_RS07805
RR41_RS19295
RR41_RS07810
RR41_RS07815
RR41_RS07820

Fig. S1g

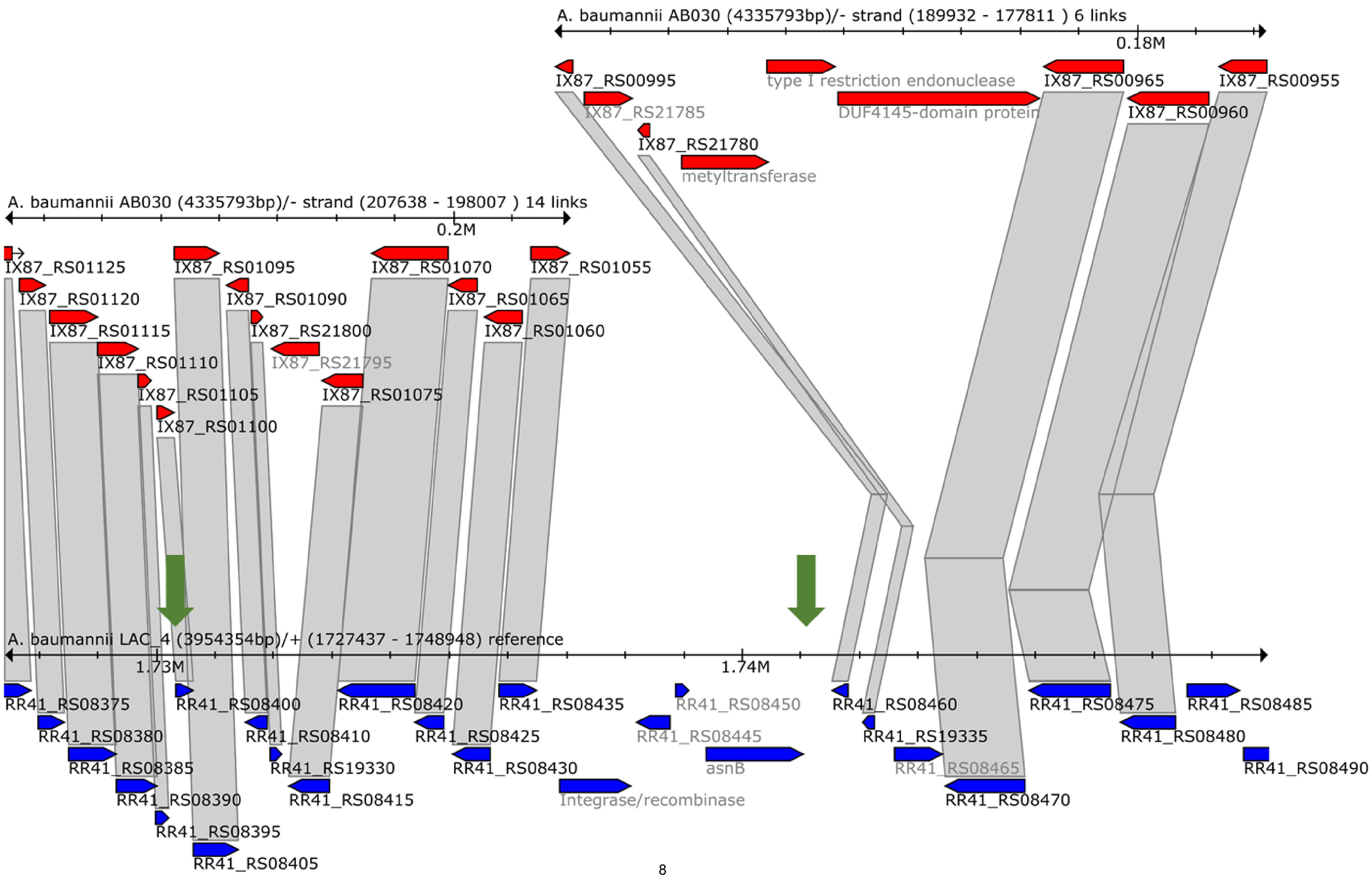
A. baumannii AB030 (4335793bp)/- strand (189932 - 177811) 6 links
0.18M
IX87_RS00995
type I restriction endonuclease
IX87_RS00965
IX87_RS00955
IX87_RS21785
DUF4145-domain protein
IX87_RS00960
IX87_RS21780
metyltransferase
A. baumannii AB030 (4335793bp)/- strand (207638 - 198007) 14 links
0.2M
IX87_RS01125
IX87_RS01095
IX87_RS01070
IX87_RS01055
IX87_RS01120
IX87_RS01090
IX87_RS01065
IX87_RS01115
IX87_RS21800
IX87_RS01060
IX87_RS01110
IX87_RS21795
IX87_RS01105
IX87_RS01075
IX87_RS01100
A. baumannii LAC_4 (3954354bp)/+ (1727437 - 1748948) reference
1.73M
1.74M
RR41_RS08375
RR41_RS08400
RR41_RS08420
RR41_RS08435
RR41_RS08450
RR41_RS08460
RR41_RS08475
RR41_RS08485
RR41_RS08380
RR41_RS08410
RR41_RS08425
RR41_RS08445
RR41_RS19335
RR41_RS08480
RR41_RS08385
RR41_RS19330
RR41_RS08430
asnB
RR41_RS08465
RR41_RS08490
RR41_RS08390
RR41_RS08415
Integrase/recombinase
RR41_RS08470
RR41_RS08395
RR41_RS08405

Fig. S1h

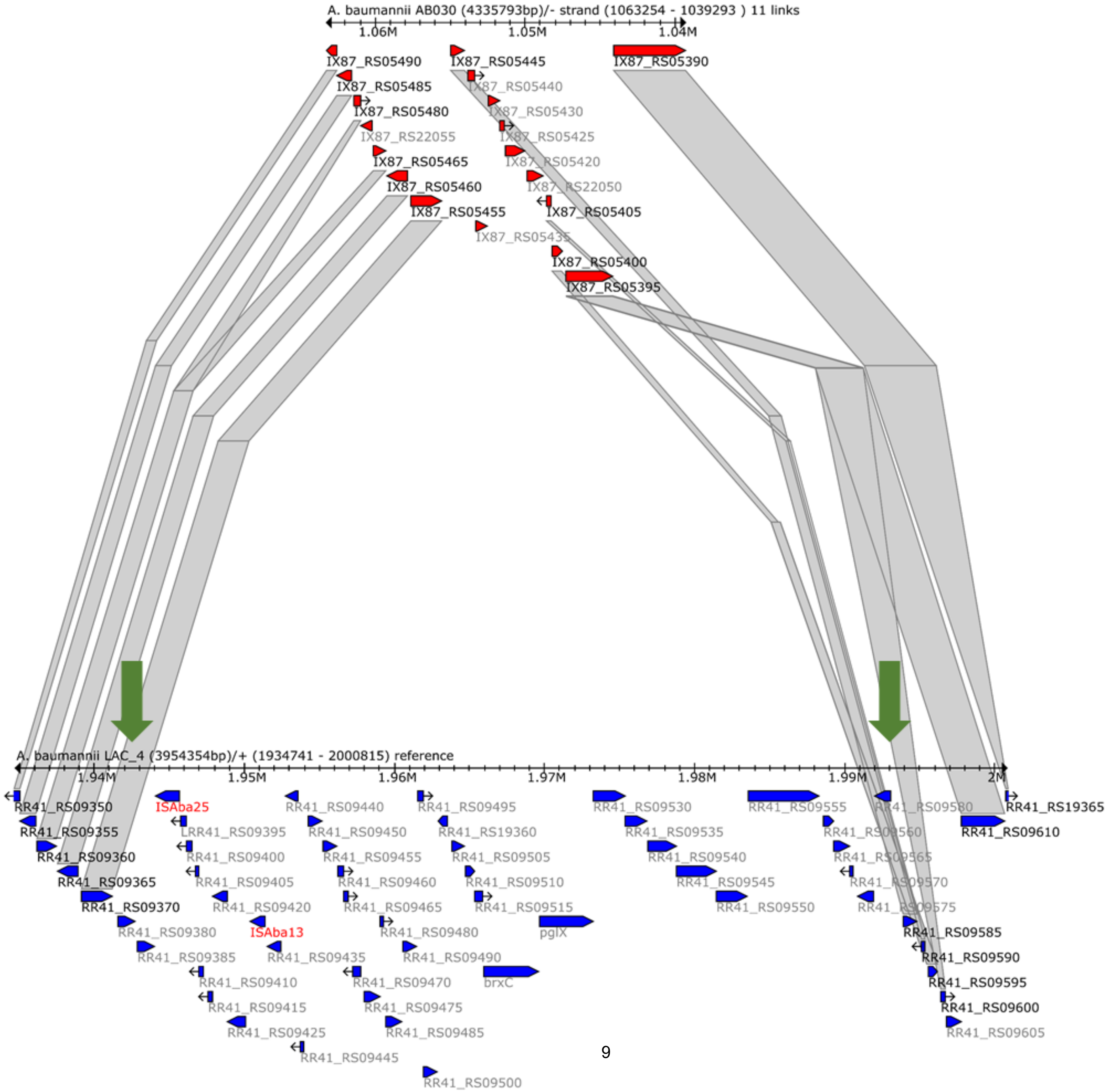
A. baumannii AB030 (4335793bp)/- strand (1063254 - 1039293) 11 links
1.06M
1.05M
1.04M
IX87_RS05490
IX87_RS05485
IX87_RS05480
IX87_RS22055
IX87_RS05465
IX87_RS05460
IX87_RS05455
IX87_RS05445
IX87_RS05440
IX87_RS05430
IX87_RS05425
IX87_RS05420
IX87_RS22050
IX87_RS05405
IX87_RS05400
IX87_RS05395
IX87_RS05390
A. baumannii LAC_4 (3954354bp)/+ (1934741 - 2000815) reference
1.94M
1.95M
1.96M
1.97M
1.98M
1.99M
2M
RR41_RS09350
RR41_RS09355
RR41_RS09360
RR41_RS09365
RR41_RS09370
RR41_RS09380
RR41_RS09385
ISAba25
RR41_RS09395
RR41_RS09400
RR41_RS09405
RR41_RS09410
RR41_RS09415
RR41_RS09420
ISAba13
RR41_RS09425
RR41_RS09435
RR41_RS09440
RR41_RS09445
RR41_RS09450
RR41_RS09455
RR41_RS09460
RR41_RS09465
RR41_RS09470
RR41_RS09475
RR41_RS09480
RR41_RS09485
RR41_RS09490
RR41_RS09495
RR41_RS19360
RR41_RS09500
RR41_RS09505
RR41_RS09510
RR41_RS09515
brxC
pglX
RR41_RS09530
RR41_RS09535
RR41_RS09540
RR41_RS09545
RR41_RS09550
RR41_RS09555
RR41_RS09560
RR41_RS09565
RR41_RS09570
RR41_RS09575
RR41_RS09580
RR41_RS09585
RR41_RS09590
RR41_RS09595
RR41_RS09600
RR41_RS09605
RR41_RS09610
RR41_RS19365

Fig. S1i

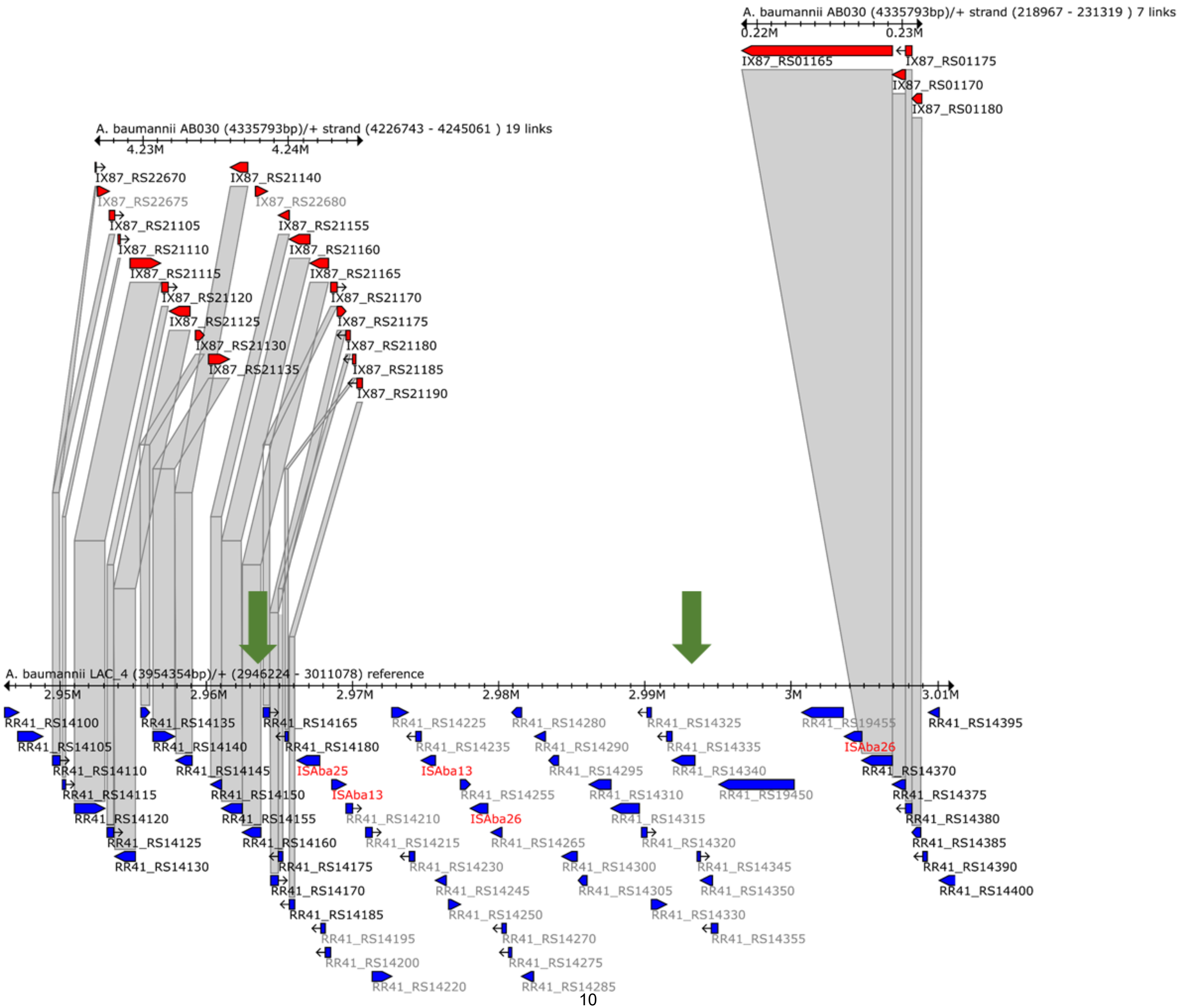
A. baumannii AB030 (4335793bp)/+ strand (218967 - 231319) 7 links
0.22M
0.23M
IX87_RS01165
IX87_RS01175
IX87_RS01170
IX87_RS01180
A. baumannii AB030 (4335793bp)/+ strand (4226743 - 4245061) 19 links
4.23M
4.24M
IX87_RS22670
IX87_RS22675
IX87_RS21105
IX87_RS21110
IX87_RS21115
IX87_RS21120
IX87_RS21125
IX87_RS21130
IX87_RS21135
IX87_RS21140
IX87_RS22680
IX87_RS21155
IX87_RS21160
IX87_RS21165
IX87_RS21170
IX87_RS21175
IX87_RS21180
IX87_RS21185
IX87_RS21190
A. baumannii LAC_4 (3954354bp)/+ (2946224 - 3011078) reference
2.95M
2.96M
2.97M
2.98M
2.99M
3M
3.01M
RR41_RS14100
RR41_RS14105
RR41_RS14110
RR41_RS14115
RR41_RS14120
RR41_RS14125
RR41_RS14130
RR41_RS14135
RR41_RS14140
RR41_RS14145
RR41_RS14150
RR41_RS14155
RR41_RS14160
RR41_RS14165
RR41_RS14180
RR41_RS14175
RR41_RS14170
RR41_RS14185
ISAba25
ISAba13
RR41_RS14195
RR41_RS14200
RR41_RS14210
RR41_RS14215
RR41_RS14220
RR41_RS14225
RR41_RS14235
ISAba13
RR41_RS14230
RR41_RS14245
RR41_RS14250
RR41_RS14255
ISAba26
RR41_RS14265
RR41_RS14270
RR41_RS14275
RR41_RS14285
RR41_RS14280
RR41_RS14290
RR41_RS14295
RR41_RS14310
RR41_RS14315
RR41_RS14300
RR41_RS14305
RR41_RS14325
RR41_RS14335
RR41_RS14340
RR41_RS14320
RR41_RS14330
RR41_RS14345
RR41_RS14350
RR41_RS14355
RR41_RS19450
RR41_RS19455
ISAba26
RR41_RS14370
RR41_RS14375
RR41_RS14380
RR41_RS14385
RR41_RS14390
RR41_RS14395
RR41_RS14400

Fig. S1j

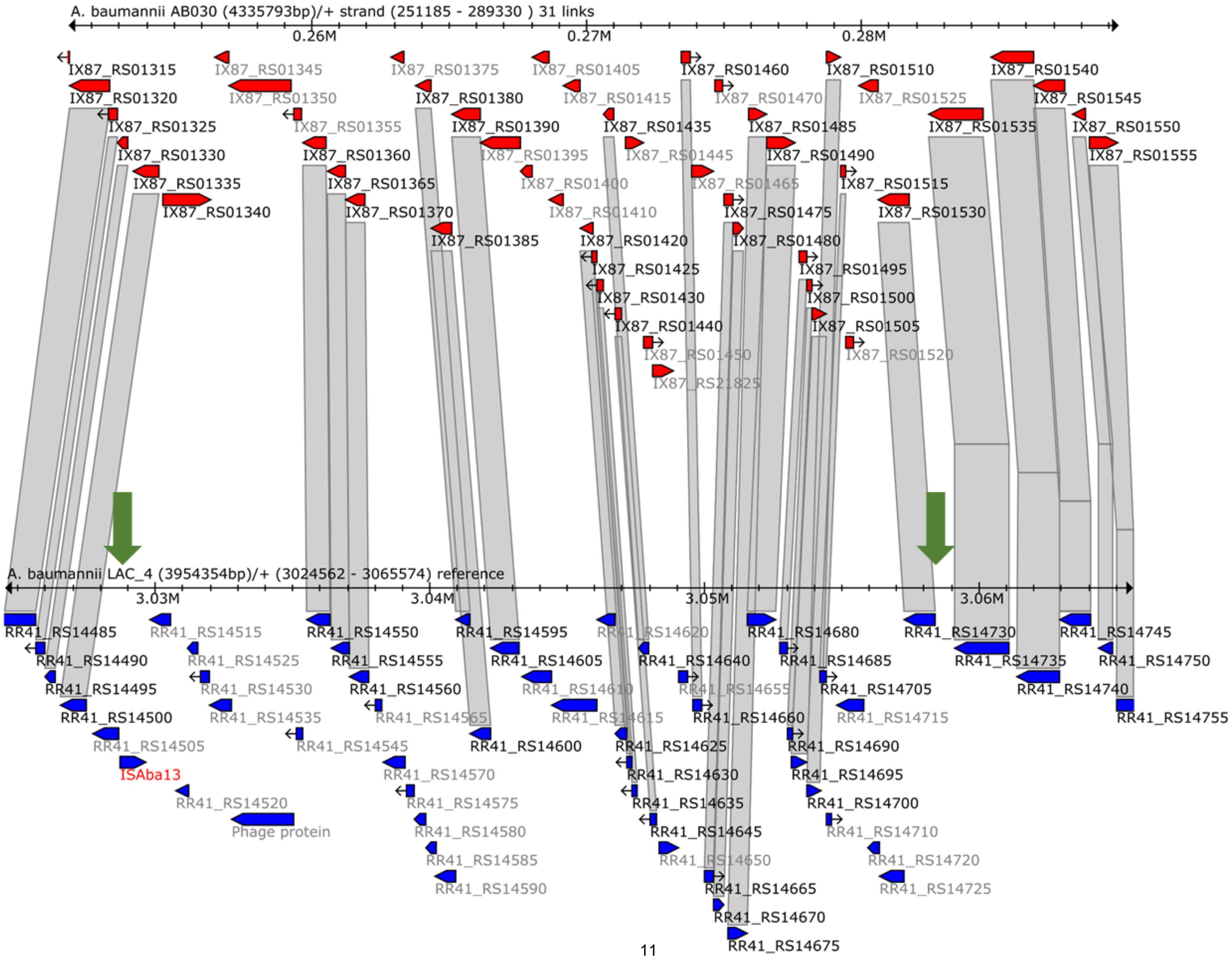
A. baumannii AB030 (4335793bp)/+ strand (251185 - 289330) 31 links
0.26M
0.27M
0.28M
IX87_RS01315
IX87_RS01320
IX87_RS01325
IX87_RS01330
IX87_RS01335
IX87_RS01340
IX87_RS01345
IX87_RS01350
IX87_RS01355
IX87_RS01360
IX87_RS01365
IX87_RS01370
IX87_RS01375
IX87_RS01380
IX87_RS01385
IX87_RS01390
IX87_RS01395
IX87_RS01400
IX87_RS01405
IX87_RS01410
IX87_RS01415
IX87_RS01420
IX87_RS01425
IX87_RS01430
IX87_RS01435
IX87_RS01440
IX87_RS01445
IX87_RS01450
IX87_RS21825
IX87_RS01460
IX87_RS01465
IX87_RS01470
IX87_RS01475
IX87_RS01480
IX87_RS01485
IX87_RS01490
IX87_RS01495
IX87_RS01500
IX87_RS01505
IX87_RS01510
IX87_RS01515
IX87_RS01520
IX87_RS01525
IX87_RS01530
IX87_RS01535
IX87_RS01540
IX87_RS01545
IX87_RS01550
IX87_RS01555
A. baumannii LAC_4 (3954354bp)/+ (3024562 - 3065574) reference
3.03M
3.04M
3.05M
3.06M
RR41_RS14485
RR41_RS14490
RR41_RS14495
RR41_RS14500
RR41_RS14505
ISAba13
RR41_RS14515
RR41_RS14520
Phage protein
RR41_RS14525
RR41_RS14530
RR41_RS14535
RR41_RS14545
RR41_RS14550
RR41_RS14555
RR41_RS14560
RR41_RS14565
RR41_RS14570
RR41_RS14575
RR41_RS14580
RR41_RS14585
RR41_RS14590
RR41_RS14595
RR41_RS14600
RR41_RS14605
RR41_RS14610
RR41_RS14615
RR41_RS14620
RR41_RS14625
RR41_RS14630
RR41_RS14635
RR41_RS14640
RR41_RS14645
RR41_RS14650
RR41_RS14655
RR41_RS14660
RR41_RS14665
RR41_RS14670
RR41_RS14675
RR41_RS14680
RR41_RS14685
RR41_RS14690
RR41_RS14695
RR41_RS14700
RR41_RS14705
RR41_RS14710
RR41_RS14715
RR41_RS14720
RR41_RS14725
RR41_RS14730
RR41_RS14735
RR41_RS14740
RR41_RS14745
RR41_RS14750
RR41_RS14755

Fig. S1k

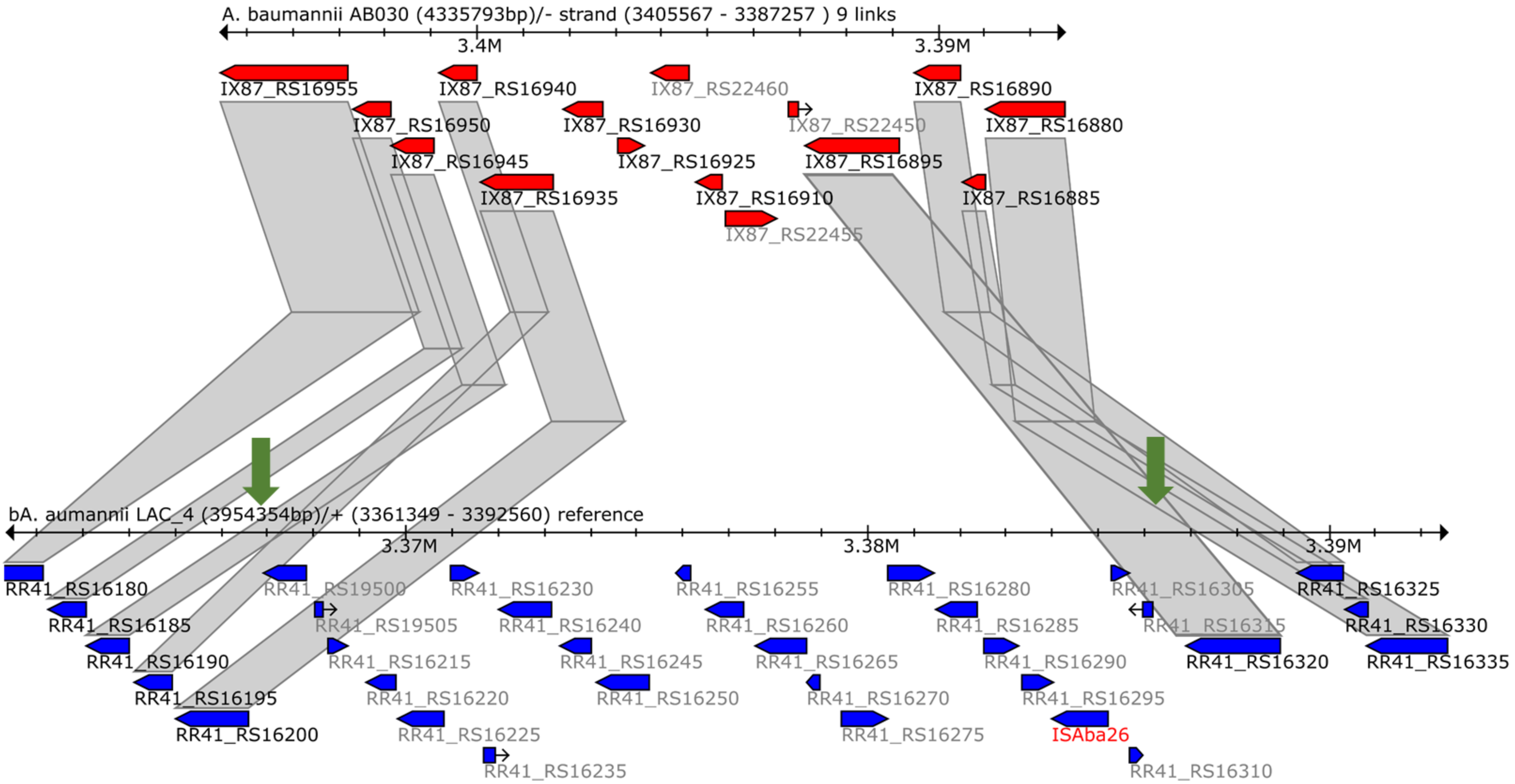
A. baumannii AB030 (4335793bp)/- strand (3405567 - 3387257) 9 links
3.4M
3.39M
IX87_RS16955
IX87_RS16940
IX87_RS22460
IX87_RS16890
IX87_RS16950
IX87_RS16930
IX87_RS22450
IX87_RS16880
IX87_RS16945
IX87_RS16925
IX87_RS16895
IX87_RS16935
IX87_RS16910
IX87_RS16885
IX87_RS22455
bA. aumannii LAC_4 (3954354bp)/+ (3361349 - 3392560) reference
3.37M
3.38M
3.39M
RR41_RS16180
RR41_RS19500
RR41_RS16230
RR41_RS16255
RR41_RS16280
RR41_RS16305
RR41_RS16325
RR41_RS16185
RR41_RS19505
RR41_RS16240
RR41_RS16260
RR41_RS16285
RR41_RS16315
RR41_RS16330
RR41_RS16190
RR41_RS16215
RR41_RS16245
RR41_RS16265
RR41_RS16290
RR41_RS16320
RR41_RS16335
RR41_RS16195
RR41_RS16220
RR41_RS16250
RR41_RS16270
RR41_RS16295
RR41_RS16200
RR41_RS16225
RR41_RS16275
ISAba26
RR41_RS16235
RR41_RS16310

Fig. S1l

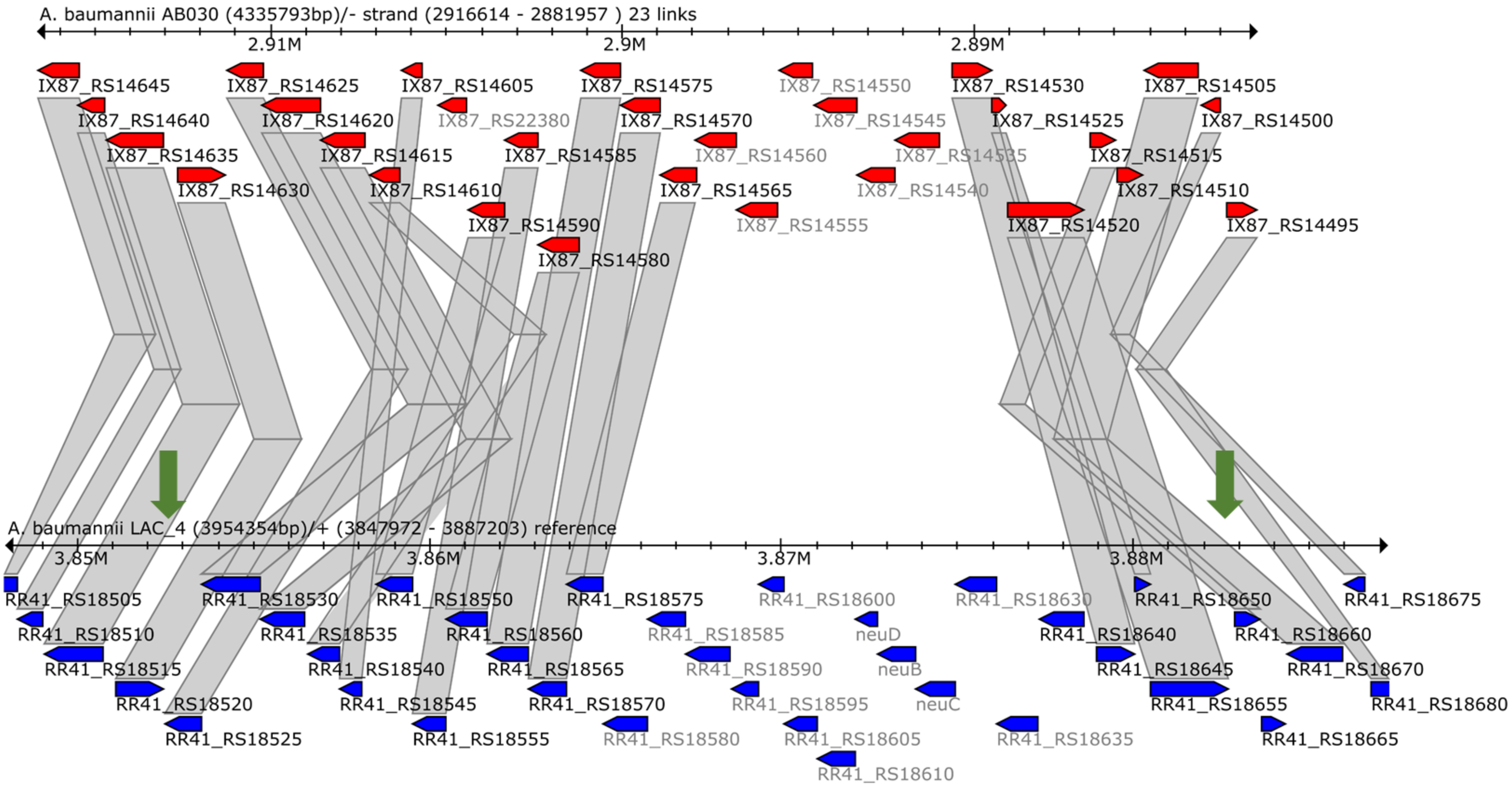
A. baumannii AB030 (4335793bp)/- strand (2916614 - 2881957) 23 links
2.91M
2.9M
2.89M
IX87_RS14645
IX87_RS14640
IX87_RS14635
IX87_RS14630
IX87_RS14625
IX87_RS14620
IX87_RS14615
IX87_RS14610
IX87_RS14605
IX87_RS22380
IX87_RS14585
IX87_RS14590
IX87_RS14580
IX87_RS14575
IX87_RS14570
IX87_RS14565
IX87_RS14560
IX87_RS14555
IX87_RS14550
IX87_RS14545
IX87_RS14540
IX87_RS14530
IX87_RS14525
IX87_RS14520
IX87_RS14515
IX87_RS14510
IX87_RS14505
IX87_RS14500
IX87_RS14495
A. baumannii LAC_4 (3954354bp)/+ (3847972 - 3887203) reference
3.85M
3.86M
3.87M
3.88M
RR41_RS18505
RR41_RS18510
RR41_RS18515
RR41_RS18520
RR41_RS18525
RR41_RS18530
RR41_RS18535
RR41_RS18540
RR41_RS18545
RR41_RS18550
RR41_RS18555
RR41_RS18560
RR41_RS18565
RR41_RS18570
RR41_RS18575
RR41_RS18580
RR41_RS18585
RR41_RS18590
RR41_RS18595
RR41_RS18600
RR41_RS18605
RR41_RS18610
neuD
neuB
neuC
RR41_RS18630
RR41_RS18635
RR41_RS18640
RR41_RS18645
RR41_RS18650
RR41_RS18655
RR41_RS18660
RR41_RS18665
RR41_RS18670
RR41_RS18675
RR41_RS18680

Fig. S2

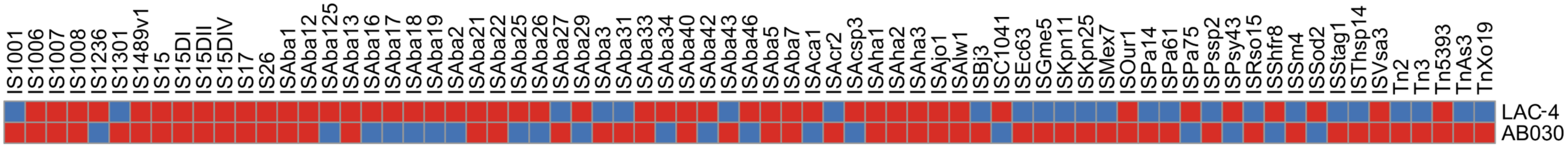
IS1001
IS1006
IS1007
IS1008
IS1236
IS1301
IS1489v1
IS15
IS15DI
IS15DII
IS15DIV
IS17
IS26
ISAba1
ISAba12
ISAba125
ISAba13
ISAba16
ISAba17
ISAba18
ISAba19
ISAba2
ISAba21
ISAba22
ISAba25
ISAba26
ISAba27
ISAba29
ISAba3
ISAba31
ISAba33
ISAba34
ISAba40
ISAba42
ISAba43
ISAba46
ISAba5
ISAba7
ISAca1
ISAcr2
ISAcsp3
ISAha1
ISAha2
ISAha3
ISAjo1
ISAlw1
ISBj3
ISC1041
ISEc63
ISGme5
ISKpn11
ISKpn25
ISMex7
ISOur1
ISPa14
ISPa61
ISPa75
ISPssp2
ISPsy43
ISRso15
ISShfr8
ISSm4
ISSod2
ISStag1
ISThsp14
ISVsa3
Tn2
Tn3
Tn5393
TnAs3
TnXo19
LAC-4
AB030

Fig. S3

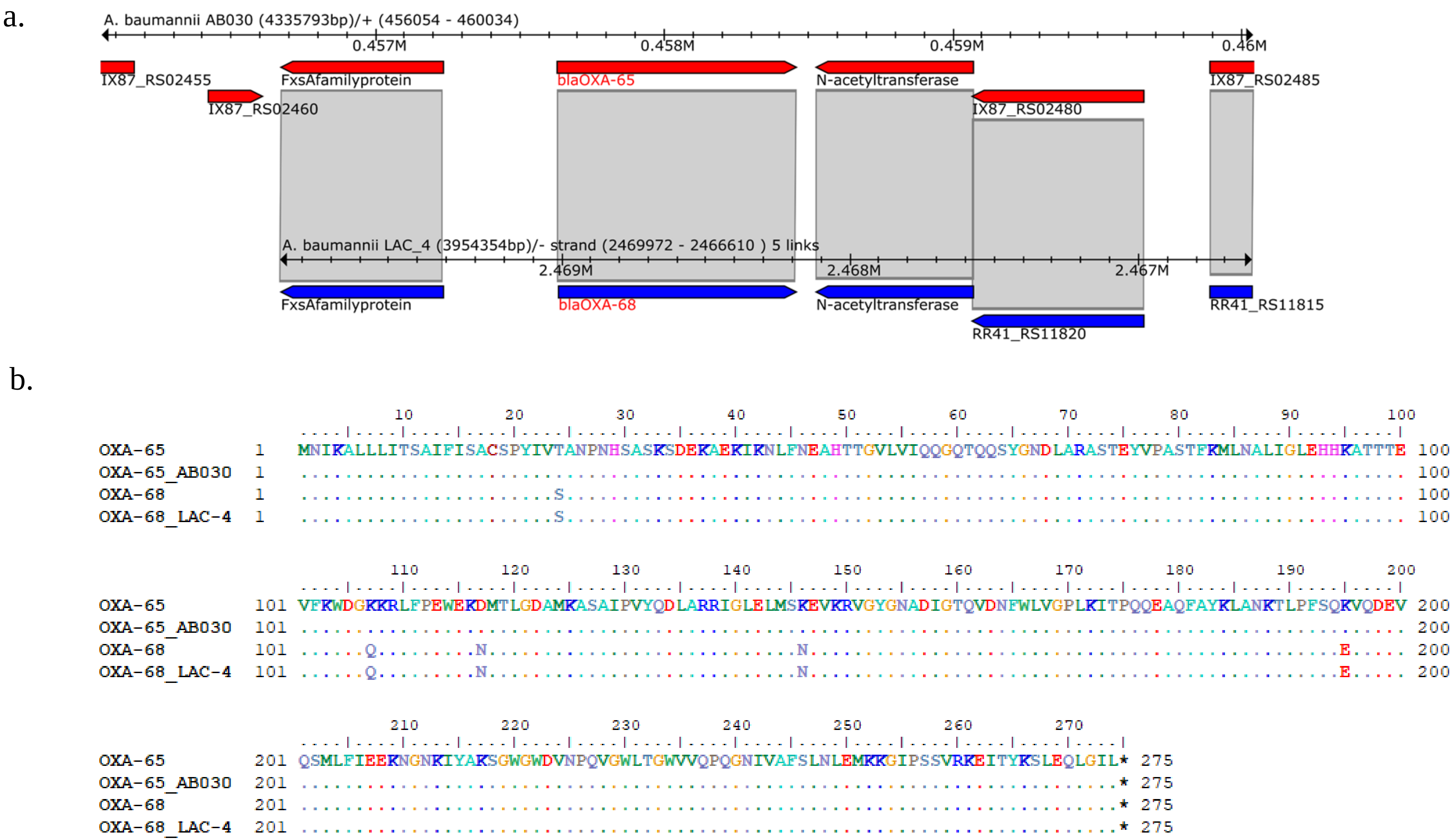
a.
A. baumannii AB030 (4335793bp)/+ (456054 - 460034)
0.457M
0.458M
0.459M
0.46M
IX87_RS02455
IX87_RS02460
FxsAfamilyprotein
blaOXA-65
N-acetyltransferase
IX87_RS02480
IX87_RS02485
A. baumannii LAC_4 (3954354bp)/- strand (2469972 - 2466610) 5 links
2.469M
2.468M
2.467M
FxsAfamilyprotein
blaOXA-68
N-acetyltransferase
RR41_RS11820
RR41_RS11815
b.
OXA-65
OXA-65_AB030
OXA-68
OXA-68_LAC-4
1 MNIKALLLITSAIFISACSPYIVTANPNHSASKSDEKAEKIKNLFNEAHTTGVLVIQQGQTQQSYGNDLARASTEYVPASTFKMLNALIGLEHHKATTTE 100
101 VFKWDGKKRLFPEWEKDMTLGDAMKASAIPVYQDLARRIGLELMSKEVKRVGYGNADIGTQVDNFWLVGPLKITPQQEAQFAYKLANKTLPFSQKVQDEV 200
201 QSMLFIEEKNGNKIYAKSGWGWDVNPQVGWLTGWVVQPQGNIVAFSLNLEMKKGIPSSVRKEITYKSLEQLGIL* 275

Fig. S4

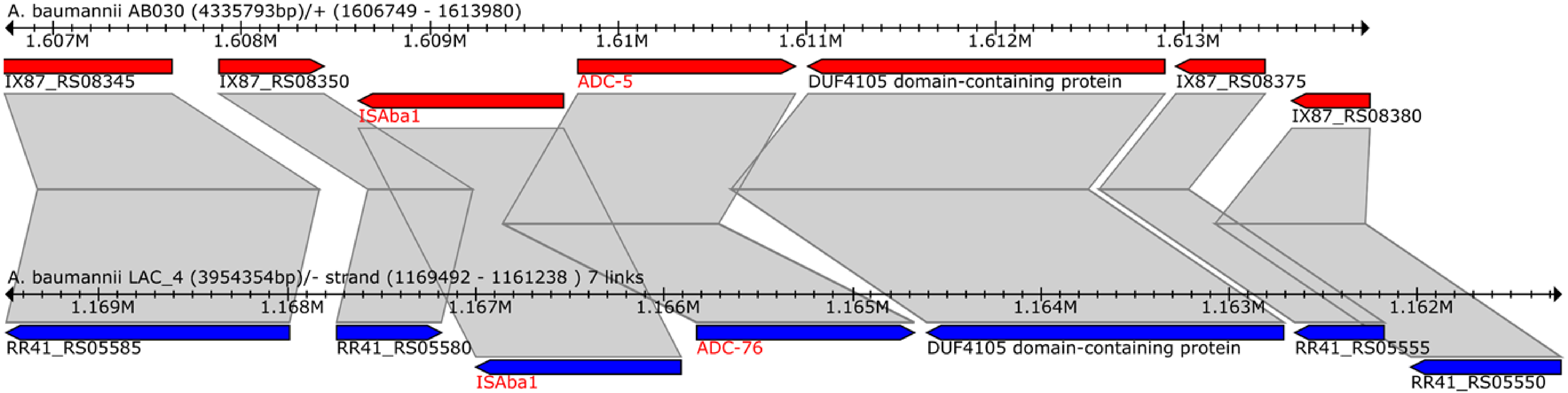

A. baumannii AB030 (4335793bp)/+ (1606749 - 1613980)
1.607M
1.608M
1.609M
1.61M
1.611M
1.612M
1.613M
IX87_RS08345
IX87_RS08350
ISAba1
ADC-5
DUF4105 domain-containing protein
IX87_RS08375
IX87_RS08380
A. baumannii LAC_4 (3954354bp)/- strand (1169492 - 1161238) 7 links
1.169M
1.168M
1.167M
1.166M
1.165M
1.164M
1.163M
1.162M
RR41_RS05585
RR41_RS05580
ISAba1
ADC-76
DUF4105 domain-containing protein
RR41_RS05555
RR41_RS05550

Fig. S5

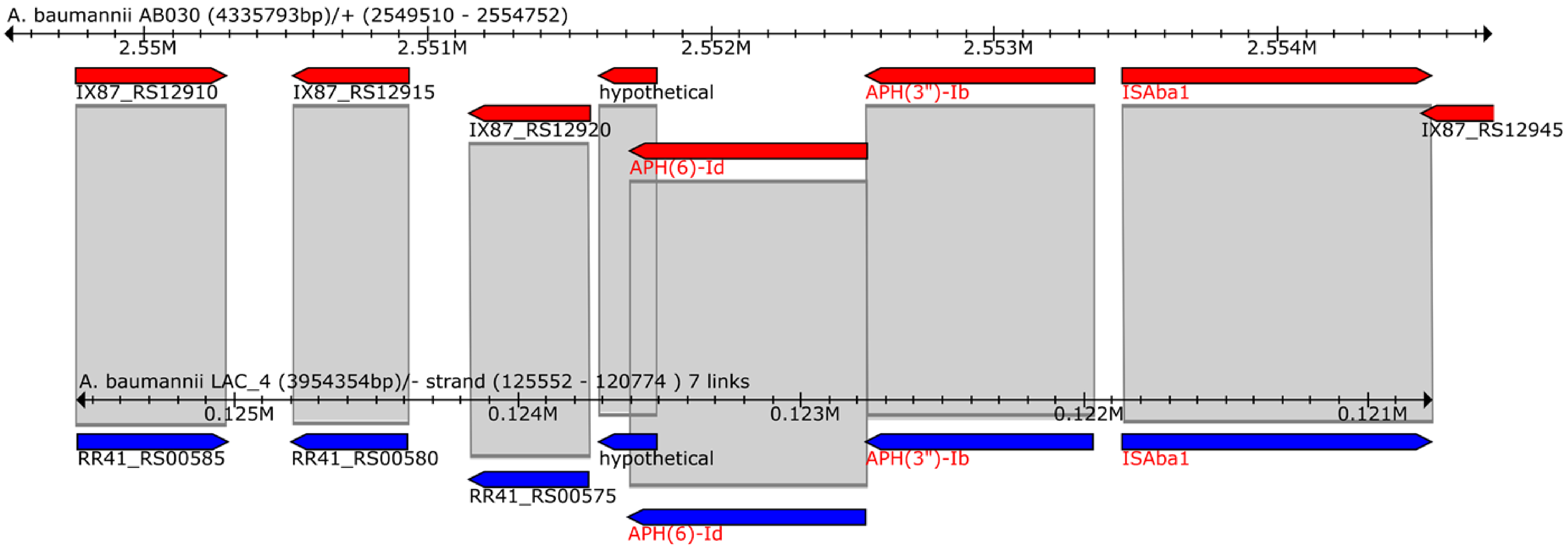
A. baumannii AB030 (4335793bp)/+ (2549510 - 2554752)
2.55M
2.551M
2.552M
2.553M
2.554M
IX87_RS12910
IX87_RS12915
hypothetical
APH(3")-Ib
ISAba1
IX87_RS12920
IX87_RS12945
APH(6)-Id
A. baumannii LAC_4 (3954354bp)/- strand (125552 - 120774) 7 links
0.125M
0.124M
0.123M
0.122M
0.121M
RR41_RS00585
RR41_RS00580
hypothetical
APH(3")-Ib
ISAba1
RR41_RS00575
APH(6)-Id

Fig. S6

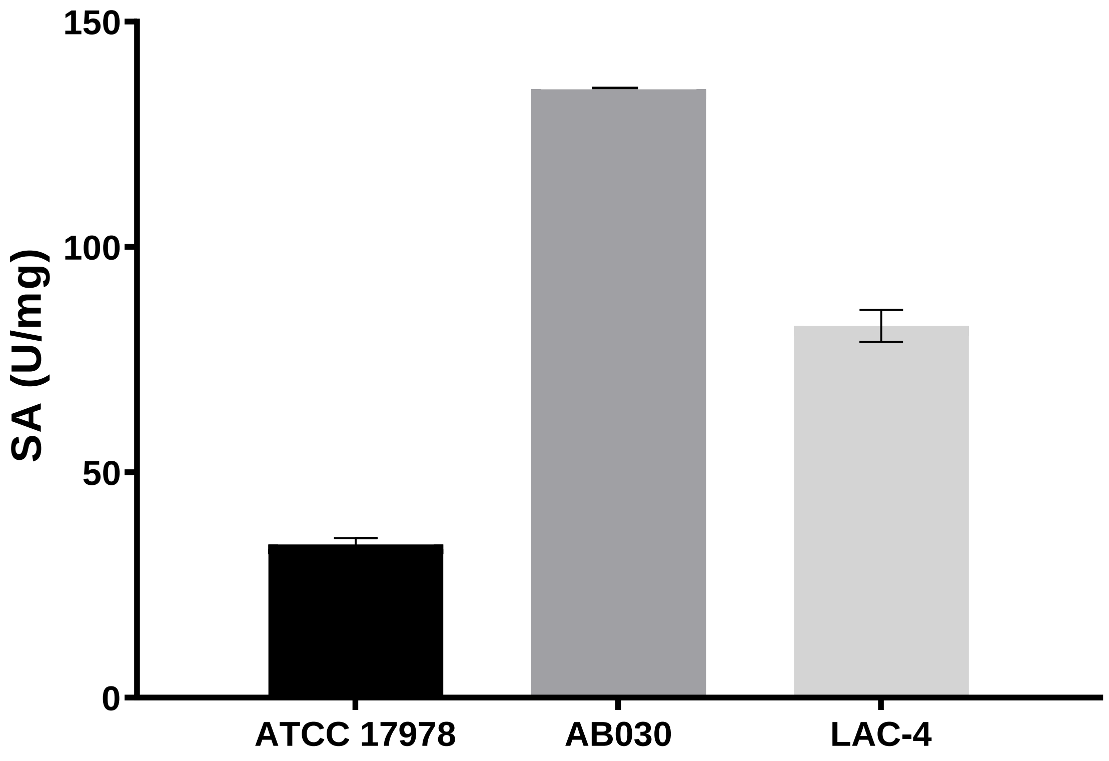

Table S1. Predicted Genomic Islands present in AB030 as identified by Islandviewer.

				In LAC-4 genome		
Island no.	Island start	Island end	Length	Query coverage	E-value	Identity
1	190685	202245	11560	38%	0	99.98%
2	268005	281740	13735	57%	0	95.69%
3*	324734	335547	10813	100%	0	99.59%
4	362940	404411	41471	24%	0	99.20%
5	476867	482761	5894	70%	0	98.84%
6	765527	804448	38921	55%	0	96.33%
7	833867	843701	9834	100%	0	97.59%
8	1032352	1039273	6921	79%	0	99.13%
9	1091422	1113975	22553	64%	0	94.74%
10	1142438	1147839	5401	47%	0	98.28%
11	1181466	1191022	9556	86%	0	98.34%
12	1487849	1496718	8869	4%	2.00E-168	90.93%
13	1546090	1563347	17257	41%	0	91.52%
14	1926318	1973925	47607	61%	0	99.38%
15	2049753	2059704	9951	12%	0	94.81%
16	2202807	2211488	8681	33%	0	99.12%
17*	2458526	2467319	8793	8%	0	99.87%
18*	2538454	2578240	39786	53%	0	99.82%
19	2658176	2665482	7306	37%	0	96.55%
20	2959816	2973667	13851	0%	0	0.00%
21	3536698	3579918	43220	66%	0	99.37%
22	3661157	3698129	36972	0%	0	0.00%
23	3855311	3859508	4197	0%	0	0.00%
24	3904427	3951898	47471	17%	0	91.59%
25	3987855	3995562	7707	15%	0	93.26%
26	4062202	4067925	5723	0%	0	0.00%
27	4305821	4328706	22885	53%	0	94.22%

*identified as resistance islands

Table S2. Predictions for the presence of phage sequences in the genomes of AB030 and LAC-4.

Phage sequences in AB030												
										In LAC-4 genome		
Region	Length	Completeness (score)	Specific keyword	Position	Total proteins	Phage proteins	Hypothetical protein	Phage + Hypothetical protein	Phage species	GC	Query coverage	E-value	Identity
1	28.6Kb	incomplete(60)	capsid, head	685-29364	54	37	15	96.20%	6	39.35%	53%	0	93.17%
2	16Kb	incomplete(60)	transposase, plate	195334-211374	19	11	8	100%	6	34.70%	80%	0	99.96%
3	9Kb	incomplete(30)	tail, transposase	241417-250505	11	7	4	100%	5	39.72%	91%	0	97.94%
4	23.1Kb	incomplete(40)	plate, transposase, integrase	263065-286249	32	17	12	90.60%	14	37.84%	74%	0	97.29%
5	53.8Kb	intact(150)	integrase, lysin, tail, head, portal	341980-395833	51	32	19	100%	21	40.15%	28%	0	98.47%
6	6.4Kb	incomplete(60)	transposase, head	576125-582561	9	7	0	77.70%	4	38.90%	75%	0	97.66%
7	49.1Kb	questionable(84)	integrase, tail, capsid, head	908821-957987	72	61	10	98.60%	16	39.85%	56%	0	88.57%
8	61.8Kb	intact(150)	integrase, transposase, tail, capsid, head	1086365-1148174	77	56	19	97.40%	17	38.82%	60%	0	93.73%
9	38.1Kb	questionable(90)	tail, capsid, head, plate	1461384-1499530	43	26	16	97.60%	17	40.81%	62%	0	95.65%
10	50.1Kb	intact(106)	integrase, tail, capsid, head, transposase	1544078-1594217	73	62	10	98.60%	12	39.29%	46%	0	91.50%
11	23.3Kb	incomplete(50)	tail, capsid, head	2036334-2059704	35	24	11	100%	13	41.75%	45%	0	95.65%
12	27Kb	intact(100)	head, portal, tail	3672382-3699422	31	24	7	100%	15	40.72%	6%	0	78.91%
13	43.2Kb	intact(150)	transposase, portal, head, tail, plate	4187963-4231200	56	41	14	98.20%	25	38.27%	12%	0	97.82%
14	17.6Kb	incomplete(30)	tail	4318067-4335678	20	19	1	100%	3	40.83%	58%	0	94.29%

Phage sequences in LAC-4													
											In AB030 genome		
Region	Length	Completeness (score)	Specific keyword	Position	Total protein	Phage protein	Hypothetical protein	Phage + Hypothetical protein	Phage species	GC	Query coverage	E-value	Identity
1	10.3Kb	incomplete(50)	transposase, head	941541-951920	11	9	0	81.80%	7	40.44%	76%	0	99.28%
2	31.2Kb	questionable(80)	head, capsid, tail	1226738-1258017	29	18	9	93.10%	13	40.63%	80%	0	95.65
3	41.5Kb	intact(100)	transposase, integrase, head, capsid, tail	1569705-1611225	62	47	12	95.10%	12	38.64%	66%	0	93.17%
4	22.1Kb	incomplete(50)	plate, transposase, integrase, lysin	1716956-1739082	12	9	3	100%	8	36.31%	85%	0	99.11%
5	10Kb	incomplete(60)	integrase, transposase	1941409-1951435	11	6	1	63.60%	3	39.55%	8%	4.00E-68	82.88%
6	29.8Kb	incomplete(20)	integrase	3028581-3058412	31	17	11	90.30%	12	35.66%	58%	0	95.64%

Table S3. Antibiotic susceptibility (µg/mL) assay of AB030 and LAC-4. Different classes of antibiotics were tested against AB030 and LAC-4 via broth dilution method. MIC values indicating resistance according to the CLSI guidelines are shown in bold.

	AMK	GEN	CHL	CRO	CIP	LVX	IPM	MEM	FEP	TGC
AB030	**64**	**>32**	1	**>64**	**>16**	**16**	**>32**	**>32**	**>64**	**>16**
LAC-4	32	**16**	1	**≥256**	**16**	≤2	≤2	4	16	≤0.25

AMK Amikacin; FEP Cefepime; CHL Chloramphenicol; CRO Ceftriaxone; CIP Ciprofloxacin; IPM Imipenem; GEN Gentamicin; LVX Levofloxacin; MEM Meropenem; TGC Tigecycline

Table S4. Presence of virulence genes in AB030 and LAC-4.

Category	Virulence factors	Related genes	LAC-4	AB030
Adherence	Outer membrane protein	*ompA*	RR41_RS03275	IX87_RS10825
Biofilm formation	PNAG (Polysaccharide poly-N-acetylglucosamine)	*pgaD*	RR41_RS06830	IX87_RS06615
		pgaC	RR41_RS06825	IX87_RS06620
		pgaB	RR41_RS06820	IX87_RS06625
		pgaA	RR41_RS06815	IX87_RS06630
	Csu pili	*csuE*	-	IX87_RS06890
		csuD	-	IX87_RS06895
		csuC	-	IX87_RS06900
		csuB	-	IX87_RS06905
		csuA	-	IX87_RS06910
		csuA/B	-	IX87_RS06915
	AdeFGH efflux pump/transport autoinducer	*adeF*	RR41_RS06070	IX87_RS07440
		adeG	RR41_RS06065	IX87_RS07445
		adeH	RR41_RS06060	IX87_RS07450
	Biofilm-associated protein	*bap*	-	IX87_RS22220*
Enzyme	Phospholipase C	*plc*	RR41_RS07450	IX87_RS14485; IX87_RS06060
	Phospholipase D	*plcD*	RR41_RS02620	IX87_RS11390
Immune evasion	Capsule	Undetermined	RR41_RS18545; RR41_RS18560; RR41_RS18650; RR41_RS18550; RR41_RS18525; RR41_RS18540; RR41_RS18635; RR41_RS18565; RR41_RS18645; RR41_RS18530; RR41_RS18640; RR41_RS18655; RR41_RS18570; RR41_RS18520; RR41_RS18625; RR41_RS18620;	IX87_RS14615; IX87_RS14605; IX87_RS14580; IX87_RS14590; IX87_RS14625; IX87_RS14610; IX87_RS14575; IX87_RS14520; IX87_RS14620; IX87_RS14560; IX87_RS14570; IX87_RS14630; IX87_RS14555; IX87_RS14565; IX87_RS14545; IX87_RS14535;

			RR41_RS18630; RR41_RS18575; RR41_RS18535; RR41_RS18610; RR41_RS18615	IX87_RS14585; IX87_RS14530; IX87_RS14540; IX87_RS14550; IX87_RS14525
	LPS	*lpsB*	RR41_RS16825	IX87_RS16395
		lpxL	RR41_RS16820	IX87_RS16400
		lpxB	RR41_RS12535	IX87_RS18825
		lpxA	RR41_RS08095	IX87_RS21220
		lpxD	RR41_RS08085	IX87_RS21230
		lpxM	RR41_RS04575	IX87_RS09335
		lpxC	RR41_RS00855	IX87_RS13380
Iron uptake	Heme utilization	Undetermined	RR41_RS08485	IX87_RS00950
		Undetermined	RR41_RS08490	IX87_RS00945
		Undetermined	RR41_RS08495	IX87_RS00940
		Undetermined	RR41_RS08500	IX87_RS00935
		Undetermined	RR41_RS08505	IX87_RS00930
		Undetermined	RR41_RS08510	IX87_RS00925
		Undetermined	RR41_RS08515	IX87_RS00920
		hemO	RR41_RS08520	IX87_RS00915
		Undetermined	RR41_RS08525	IX87_RS00910
		Undetermined	RR41_RS08530	IX87_RS00905
	Acinetobactin	*basJ*	RR41_RS05535	IX87_RS08395
		basI	RR41_RS05530	IX87_RS08400
		basH	RR41_RS05525	IX87_RS08405
		barB	RR41_RS05520	IX87_RS08410
		barA	RR41_RS05515	IX87_RS08415
		basG	RR41_RS05505	IX87_RS08425
		basF	RR41_RS05500	IX87_RS08430
		entE	RR41_RS05495	IX87_RS08435
		basD	RR41_RS05490	IX87_RS08440
		basC	RR41_RS05485	IX87_RS08445
		bauA	RR41_RS05480	IX87_RS08450
		bauB	RR41_RS05475	IX87_RS08455
		bauE	RR41_RS05470	IX87_RS08460
		bauC	RR41_RS05465	IX87_RS08465
		bauD	RR41_RS05460	IX87_RS08470

		basB	RR41_RS05455	IX87_RS08475
		basA	RR41_RS05450	IX87_RS08480
		bauF	RR41_RS05445	IX87_RS08485
Regulation	Quorum sensing	*abaI*	-	IX87_RS14815
		abaR	-	IX87_RS14825
	Two-component system	*bfmR*	RR41_RS15530	IX87_RS17690
		bfmS	RR41_RS15525	IX87_RS17695
Serum resistance	PbpG	*pbpG*	RR41_RS17785	IX87_RS15425
Stress adaptation	Catalase	*katA*	-	IX87_RS20175

*detected using BLASTn.
